# Autophagy-Associated Protein SmATG12 Is Required for Fruiting-Body Formation in the Filamentous Ascomycete *Sordaria macrospora*

**DOI:** 10.1371/journal.pone.0157960

**Published:** 2016-06-16

**Authors:** Antonia Werner, Britta Herzog, Stefan Frey, Stefanie Pöggeler

**Affiliations:** 1 Institute of Microbiology and Genetics, Department of Genetics of Eukaryotic Microorganisms, Georg-August University, Göttingen, Germany; 2 Göttingen Center for Molecular Biosciences (GZMB), Georg-August University, Göttingen, Germany; The University of Wisconsin - Madison, UNITED STATES

## Abstract

In filamentous fungi, autophagy functions as a catabolic mechanism to overcome starvation and to control diverse developmental processes under normal nutritional conditions. Autophagy involves the formation of double-membrane vesicles, termed autophagosomes that engulf cellular components and bring about their degradation via fusion with vacuoles. Two ubiquitin-like (UBL) conjugation systems are essential for the expansion of the autophagosomal membrane: the UBL protein ATG8 is conjugated to the lipid phosphatidylethanolamine and the UBL protein ATG12 is coupled to ATG5. We recently showed that in the homothallic ascomycete *Sordaria macrospora* autophagy-related genes encoding components of the conjugation systems are required for fruiting-body development and/or are essential for viability. In the present work, we cloned and characterized the *S*. *macrospora (Sm)atg12* gene. Two-hybrid analysis revealed that SmATG12 can interact with SmATG7 and SmATG3. To examine its role in *S*. *macrospora*, we replaced the open reading frame of *Smatg12* with a hygromycin resistance cassette and generated a homokaryotic ΔSmatg12 knockout strain, which displayed slower vegetative growth under nutrient starvation conditions and was unable to form fruiting bodies. In the hyphae of *S*. *macrospora* EGFP-labeled SmATG12 was detected in the cytoplasm and as punctate structures presumed to be phagophores or phagophore assembly sites. Delivery of EGFP-labelled SmATG8 to the vacuole was entirely dependent on SmATG12.

## Introduction

In eukaryotes, macroautophagy (hereafter autophagy) is a highly conserved degradation process by which cytoplasmic components—such as organelles—are non-selectively engulfed by double-membrane vesicles called autophagosomes. After fusion of the autophagosomal outer membrane with the vacuole/lysosome, vesicles surrounded by the inner membrane of the autophagosome are released into the lumen of the vacuole. These vesicles or autophagic bodies are degraded by hydrolytic enzymes into building blocks that are recycled and released back into the cytoplasm [1]. Initially perceived as a cellular adaption to survive starvation conditions, it is now accepted that autophagy is associated with differentiation processes and various diseases in multicellular eukaryotes [2,3]. The autophagic process can be divided into five steps: induction, nucleation, elongation and closure, fusion with the vacuole, and breakdown of autophagic bodies. This process has been studied intensively in the unicellular yeast *Saccharomyces cerevisiae*. To date, 40 autophagy-associated (*atg)* genes have been discovered in yeast through genetic screening [4–6], and many homologs have been identified in multicellular eukaryotes including mammals, plants and filamentous fungi [7–13]. Of these, eight proteins are involved in two ubiquitin-like (UBL) conjugation systems that are essential for autophagosome formation: the conjugation of the UBL protein ATG8 to the lipid phosphatidylethanolamine (PE) and the conjugation of the UBL protein ATG12 to ATG5 [14].

ATG8 is initially processed to a glycine-exposed form by the protease ATG4, then activated in an ATP-dependent manner by the E1-like enzyme ATG7, subsequently transferred to the E2-like enzyme ATG3, and finally conjugated to the amino group of PE [15–19]. ATG12 is also activated by the E1-like enzyme ATG7 but is transferred to the E2-like conjugating enzyme ATG10, which covalently attaches it to a lysine residue of ATG5 [15,18]. The resulting ATG12~ATG5 conjugate then forms a complex with the coiled-coil protein ATG16, and this complex acts as an ubiquitin ligase-like E3 enzyme for the ATG8-PE conjugation reaction by stimulating the activity of ATG3 and promoting the transfer of ATG8 from ATG3 to the PE substrate [20–22]. Both UBL conjugates (ATG8-PE and the ATG12~ATG5-ATG16 complex) are localized to autophagosome precursor membranes known as pre-autophagosomal structures or phagophore assembly sites (PAS) [23]. In *S*. *cerevisiae*, ATG8-PE recruits the ATG12~ATG5-ATG16 complex for membrane localization by recognizing a non-canonical ATG8-interacting motif (AIM) in ATG12 [24]. In the growing phagophore the ATG12~ATG5-ATG16 complex localizes exclusively to the convex side of the growing cup-shaped phagophores, while ATG8-PE localizes at both sides of the phagophore [25,26]. On the convex membrane site of the phagophores, ATG16 promotes oligomerization of the ATG12~ATG5-ATG16 complex and assembles with ATG8-PE into a two-dimensional meshwork [24]. In addition to its functions as an E3-ligase and membrane scaffold, mammalian ATG12 is reported to be involved in mitochondrial homeostasis, impaired endosomal trafficking, and cell death, following conjugation to the E2-like enzyme ATG3 [27,28]. Additionally, mammalian ATG12 functions as a positive regulator of mitochondrial apoptosis by binding to and inhibiting anti-death proteins via its BH3 domain [29].

Autophagy is required for the development of fruiting bodies in the filamentous ascomycete *Sordaria macrospora*, a model organism for investigating multicellular sexual development in fungi [30–35]. Fruiting bodies of *S*. *macrospora* are known as perithecia which contain ~150 meiosporangia or asci that are produced within 7 days following germination of a sexual ascospore. Within 3 days, the mycelium derived from the germinating ascospore forms female gametangia (ascogonia). These are enwrapped by sterile hyphae and form spherical pre-fruiting bodies (protoperithecia). Self-fertilization and cellular differentiation events then lead to formation of an outer pigmented peridial tissue and inner ascus initials. Meiosis and a postmeiotic mitosis give rise to eight linearly-ordered ascospores per ascus. After maturation black-pigmented ascospores are forcibly discharged from the perithecium [36].

Surprisingly, we were not able to generate a homokaryotic ΔSmatg7 mutant in *S*. *macrospora*, suggesting that *Smatg7* encoding the E1 enzyme of both conjugation systems is required for viability [37]. However, homokaryotic deletion mutants of *Smatg8* and *Smatg4* were generated. Both mutants displayed impaired vegetative growth, and development was arrested at the pre-fruiting body stage [34]. *S*. *macrospora* is therefore a good model system to study the impact of autophagy on fungal fruiting-body development [32]. Complementation studies in *S*. *cerevisiae* demonstrated that the *S*. *macrospora* genes *Smatg7*, *Smatg8* and *Smatg4* are able to functionally replace their yeast homologs [34,37]. Furthermore, we demonstrated that the protease SmATG4 is capable of processing the SmATG8 precursor. SmATG8 localizes to autophagosomes, whereas SmATG4 is distributed throughout the cytoplasm of *S*. *macrospora* [34].

In this study, we isolated the *S*. *macrospora Smatg12* gene that encodes the second UBL autophagy-associated protein and used targeted gene replacement to analyze its role in vegetative growth and fruiting-body development. The ΔSmatg12 mutant displayed arrested sexual development at the protoperithecia-formation stage and was therefore unable to produce fruiting bodies and ascospores. Our data show that the degradation of cell components by autophagy play important roles in vegetative growth and sexual reproduction of filamentous ascomycetes and is therefore relevant for understanding fungal development.

## Material and Methods

### Strains and culture conditions

All strains used in this study are listed in Table A in S1 File. Standard protocols were used for the cultivation of *Escherichia coli* and *S*. *cerevisiae* [38,39]. *S*. *macrospora* strains were cultivated either on solid or liquid corn meal medium (biomalt maize medium, BMM), solid complex rich medium (CMS: 1% (w/v) glucose, 0.2% (w/v) tryptone/peptone, 0.2% (w/v) yeast extract, 0.15% (w/v) KH_2_PO_4_, 0.05% (w/v) KCl, 0.05% (w/v) MgSO_4_ heptahydrate, 0.37% (w/v) NH_4_Cl, 10.8% (w/v) sucrose, 0.01% (v/v) trace-element stock solution (10 mg/l ZnSO_4_, 10 mg/l Fe(II)Cl_2_, 10 mg/l MnCl_2_), pH 6.5; 1.5% (w/v) agar-agar for solid medium) or on solid synthetic fructification medium (Sordaria Westergaard’s medium, SWG: 2% glucose (w/v), 0.1% arginine (w/v) and 0.1% biotin (v/v) and Westergaard´s solution (0.1% (w/v) KNO_3_, 0.1% (w/v) KH_2_PO_4_, 0.05% (w/v) MgSO_4_ heptahydrate, 0.01% (w/v) NaCl, 0.01% (w/v) CaCl_2_, 0.01% (v/v) trace-element stock solution [5% (w/v) citric acid (C_6_H_8_O_7_ monohydrate), 5% (w/v) ZnSO_4_ heptahydrate, 1% (w/v) Fe(NH_4_)_2_(SO_4_)_2_ hexahydrate, 0.25% (w/v) CuSO_4_ pentahydrate, 0.05% (w/v) MnSO_4_ monohydrate, 0.05% (w/v) H_3_BO_3_, 0.05% (w/v) Na_2_MoO_4_ dihydrate], 0.1% (v/v) chloroform, pH 6.5; 1.5% (w/v) agar-agar for solid medium) [40,41] at 27°C or supplemented with the autophagy inducing agent 2.5 mM 3-Amino-1,2,4-triazole (3-AT). Nitrogen starvation conditions were induced on SWG medium without KNO_3_ and arginine. For analysis of the growth velocity, 30-cm race tubes were filled with 25 ml of solid SWG medium and inoculated with a mycelia plug of 0.5 cm in diameter at one end. The growth front was marked every 24 h for seven consecutive days. The growth rate was calculated in growth rate per day. For examination of the foraging abilities of *S*. *macrospora* strains, a plug test was performed according to Josefsen et al. [42]. An agar plug with a diameter of 0.5 cm was put into a cell-culture plate (6 well, 17.2 ml) and incubated for five days in a damp chamber at 27°C.

### Transformation techniques and plasmid construction

Transformation of chemically competent *E*. *coli* MACH1 cells was achieved by standard transformation protocols [39]. *S*. *cerevisiae* strain PJ69-4A [43] was used as host for homologous recombination experiments performed according to Colot et al. [44]. The transformation was performed using the electroporation method with an Eppendorf Electroporator 2510 (Eppendorf) at 1.5 kV [45] or with the lithium acetate method as described by Ito et al. [46]. Standard transformation protocols for *S*. *macrospora* were conducted as described previously [41]. Transformants were selected on media containing nourseothricin-dihydrogen sulfate (50 μg/ml) (WernerBioAgents, 5004000) or hygromycin B (110 U/ml) (Merck, 400051-10MU). Plasmids generated for this study were constructed by homologous recombination in *S*. *cerevisiae* or by ligation of fragments into cloning vectors [39,44]. All plasmids used in this study are listed in Table B in S1 File.

### Preparation of nucleic acids, PCR and RT-PCR

Genomic DNA (gDNA) of *S*. *macrospora* was isolated as described previously [47]. The extraction of RNA was done using triazol according to Elleuche and Pöggeler [48]. Reverse transcription of 1 μg RNA was performed as described by Pöggeler et al. [49]. The “Transcriptor High Fidelity cDNA Synthesis Kit” (Roche, 05081955001) was used for the reverse transcription reaction to synthesize cDNA. PCR amplification of gDNA and cDNA was performed with the “Phusion High-Fidelity DNA polymerase” (Thermo Scientific) as described in the manufacturer’s manual. *S*. *cerevisiae* gDNA isolation was performed as described by Hoffman and Winston [50]. Primers were synthesized by Eurofins (Ebersberg, Germany) and are listed in Table C in S1 File.

### Light and fluorescence microscopic investigations

For light and fluorescence microscopic analysis, *S*. *macrospora* strains were grown on solid medium on top of a sheet of cellophane (1 cm x 1 cm) in petri dishes at 27°C for 24 h. Glass slides were prepared with mycelium-coated cellophane. This was placed upside-down and covered with water and a cover slip for microscopic analysis. Fluorescence microscopic investigations were carried out with an “AxioImager M1 microscope” (Zeiss, Jena, Germany) and images were captured with a “Photometrix CoolSNAP HQ camera” (Roper Scientific, Photometrics, Tucson, USA) and processed with the programs MetaMorph (version 6.3.1; Universal Imaging) and ImageJ (Image Processing and Analysis in Java). The “chroma filter set 49002” (exciter ET470/40x, emitter ET525/50 m and beamsplitter T495LP) was used to visualize EGFP fluorescence. For phenotypic analysis *S*. *macrospora* strains were grown on glass slides covered with solid SWG medium at 27°C with continuous light. The growth period differed between 3–8 days depending on the developmental stage. Later stages of fruiting-body development were captured with the “Digital Microscope VHX-500F” (Keyence, Germany). Mycelial growth in the foraging test was visualized using a “Motic SMZ-168 stereo zoom microscope” (Ted Pella, Inc, USA).

For localization of SmATG12 an *egfp*-tagged variant of *Smatg12* under control of the native promoter and terminator was constructed. The promoter region was amplified from wt gDNA using primer pair Atg12-gfp-5f/Atg12-gfp-5r (1058 bp) and the coding region including the 3´ region of *Smatg12* (1615 bp) with primer pair Atg12-gfp-3f2/Atg12-gfp-3r (Table C in S1 File). The primers exhibited 29-bp overhangs to the neighboring regions for homologous recombination reactions. The *egfp* was amplified from plasmid p1783-1 [51] using primer pair GFP-f/GFP-r (688 bp). All fragments were subcloned into the *Xho*I-linearized vector pRSnat [52]and resulted in N-terminally *egfp*-tagged version of *Smatg12* in plasmid pegfp-Smatg12 (Table B in S1 File). Staining of vacuolar membranes was achieved by applying 50–100 μl of a FM4-64 solution (Invitrogen, F34653; 1 μg/ml in dH_2_O) directly on the mycelium and incubation for 15 min. FM4-64 fluorescence was recorded with a “chroma filter set 49005” (excitation/emission filter ET545/30/ET620/60, beam splitter T570lp) and an X-cite 120 PC lamp (EXFO).

### Sequence analysis

Protein sequence alignments were done using the ClustalX program [53] with ATG12, ATG3 and ATG5 sequences of different organisms obtained after BLASTP search from the public databases at NCBI (http://www.ncbi.nlm.nih.gov/entrez/). Molecular weights and isoelectric points of proteins were calculated with programs from the ExPASy Proteomics Server (http://www.expasy.org). DNA sequencing was performed by the G2L-sequencing service of the “Göttinger Genom Labor” (Georg-August University of Göttingen, Germany).

### Construction of knockout strain ΔSmatg12

To delete the *Smatg12* gene in *S*. *macrospora*, a knockout construct was generated. The 5’- (1058 bp) and 3’- (700 bp) flanking regions of *Smatg12* were amplified from *S*. *macrospora* wt gDNA using the primer pairs Atg12_5f/Atg12_5r and Atg12_3f/Atg12_3r carrying 29-bp overhangs for the pRS426 vector [54] and the hygromycin resistance B (*hph)* cassette, respectively. The *hph*-cassette (1419 bp) was amplified from plasmid pCB1003 [55] with the primers hph-f/hph-r. Subsequently, the three amplicons were co-transformed together with the *Xho*I-linearized vector pRS426 into the yeast strain PJ69-4A. Transformants were selected on selective dropout (SD)-medium lacking uracil. The recombinant plasmid pSmatg12-KO, consisting of the upstream and downstream sequence of *Smatg12* interrupted by the *hph*-cassette, was isolated from yeast as described by Hoffman and Winston [50]. Plasmid pSmatg12-KO served as template to generate the 3235-bp knockout fragment by PCR with primer pair Atg12_5f/Atg12_3r. The amplicon was desalted and then transformed into *S*. *macrospora* Δku70 strain [56] to facilitate the *Smatg12* knockout by homologous recombination. Hygromycin B resistant primary transformants were analyzed by PCR with primer pairs Atg12_3D1/h3 and tC1/Atg12_5D1 to verify the homologous recombination event at the desired *Smatg12* gene locus. Primary transformants of *S*. *macrospora* are usually heterokaryotic carrying Δku70 (nat^R^) and mutant nuclei (nat^R^/hyg^R^). To obtain homokaryotic deletion mutants and to eliminate the Δku70 (nat^R^) background, primary transformants were crossed with the brown spore-color mutant fus1-1 [57]. In in the homothallic *S*. *macrospora*, recombinant hybrid perithecia can be easily identified when the crossing partners differ in spore color. The respective strains were inoculated directly towards each other on a petri dish with solid SWG medium. The plates were incubated for 10–12 days at 27°C until a crossing front was formed in the middle of the petri dish. This crossing front contained the recombinant hybrid perithecia with asci containing 4 brown and 4 black ascospores in typical 4:4 or 2:2:2:2 segregation patterns [58].

Spores from hybrid perithecia were isolated and selected on BMM agar plates containing only hygromycin B (110 U/ml) and 0.5% sodium acetate. The resulting homokaryotic deletion strain ΔSmatg12 was tested by PCR for the absence of the *Smatg12* gene using primer pair Atg12_5D1/Atg12_3D2. gDNA of *S*. *macrospora* wt strain was used as control. Deletion of *Smatg12* was verified by Southern hybridization [59]. Therefore, 30–50 μg of gDNA was hydrolyzed with *Bgl*I and separated on an 1% agarose gel. A semi-dry blot onto a nitrocellulose membrane (GE Healthcare, Amersham, 10600003, Germany) was performed for 3 h. The “AlkPhos Direct Labelling and Detection Kit” (GE Healthcare, Germany) was used for labelling the 300-bp probe amplified with primer pair Atg12_PRf/Atg12_PRr from *S*. *macrospora* genomic DNA and purified with QIAquick gel extraction kit (Qiagen, Hilden, RPN3690, Germany). Detection was performed according to the manufacturer’s manual.

### Yeast complementation

To analyze functional conservation of *atg12* in *S*. *macrospora* and *S*. *cerevisiae* the aminopeptidase 1 (Ape1) maturation assay was carried out as described by Harding et al. [60]. The 480-bp *Smatg12* cDNA amplified with primer pair Atg12_Cf/Atg12_Cr having *Spe*I and *Sal*I overhangs, respectively, was expessed under the control of the yeast *MET25* promoter in pRS-met25-Smatg12 [61]. Plasmid pRS-met25-Scatg12 was generated by cloning a 561-bp *Scatg12* fragment amplified from *S*. *cerevisiae* BY4741 wt gDNA into the *Eco*RI/*Spe*I-hydrolyzed plasmid pRS426-met25. In the next step, yeast strain Y03357 (atg12Δ) was transformed with plasmids pRS-met25-Smatg12 and pRS-met25-Scatg12. As negative controls, the *S*. *cerevisiae* wt strain BY4741 as well as the yeast atg12Δ deletion strain, were transformed with the empty vector pRS426-met25 [61]. To test rescue of the Ape1 processing in the atg12Δ mutant, cells were grown over night in SD minimal medium and adjusted to OD_600_ = 1. One set of cells was used for protein extraction, while another set was grown for four hours in SD minimal medium lacking nitrogen (SD-N) to induce amino-acid starvation and, in turn, autophagy in *S*. *cerevisiae* [60]. Details of the protein extraction and Western Blot analysis are described in Method A S1 File.

### Yeast-two hybrid interaction

The construction of the two-hybrid plasmids used in this study and verification of the expression of the GAL4 fusion proteins is described in Method B S1 File.

*S*. *cerevisiae* strains AH109 and Y187 were used for yeast-two hybrid experiments. Mat α strain Y187 was transformed with bait plasmids pGBKT7, pBD-Smatg8, pBD-Smatg12, pBD-Smatg3 or pBD-Smatg7 and transformants were selected for tryptophan prototrophy, whereas Mat a strain AH109 was transformed with prey plasmids pGADT7, pAD-ranBPM, pAD-Smatg8, pAD-Smatg12, pAD-Smatg3 or pAD-Smatg7 and transformants were selected for leucine prototrophy. Recombinant AH109 and Y187 strains were mated and selected on solid SD minimal medium lacking both, tryptophan and leucine. Alternatively, both plasmids were co-transformed into strain AH109. Interaction of the bait and prey fusion constructs was confirmed by growth on selective SD minimal medium lacking histidine and adenine. Cells grown to the log phase in liquid SD minimal medium without leucine and tryptophan were diluted to an optical density (OD) of 0.1. From this main dilution, 20 μl were spotted in a 1:10 dilution series onto selective SD plates, which were incubated at 30°C for five days. Yeast growth was visualized using a “Digital Microscope VHX-500F” (Keyence, Germany).

### *S*. *macrospora* protein extaction

For detection of EGFP-SmATG8 fusion proteins *S*. *macrospora* protein extracts were prepared similar to Bloemendal et al. [62]. The lysis buffer (600 μl/g dried mycelium: 10 mM Tris (pH7.5), 150 mM NaCl, 0.5 mM EDTA (pH 8.0), 2 mM DTT, 1 mM PMSF, 0.5x protease inhibitor cocktail, 10% glycerol, 0.5% NP40; glass sand) was modified. Immunodetection of the fusion protein was achieved with a monoclonal rat anti-EGFP antibody (1:4000, Chromotek 3H9 029762) and a secondary antibody a goat anti-rat HRP-linked antibody (1:5500, Invitrogen 62–9520) was used. As loading control the actin protein was detected as described in Method A S1 File.

## Results

### Identification of an *S*. *macrospora* Atg12 homolog as an interaction partner of SmATG7 and SmATG3

**Fig 1:**
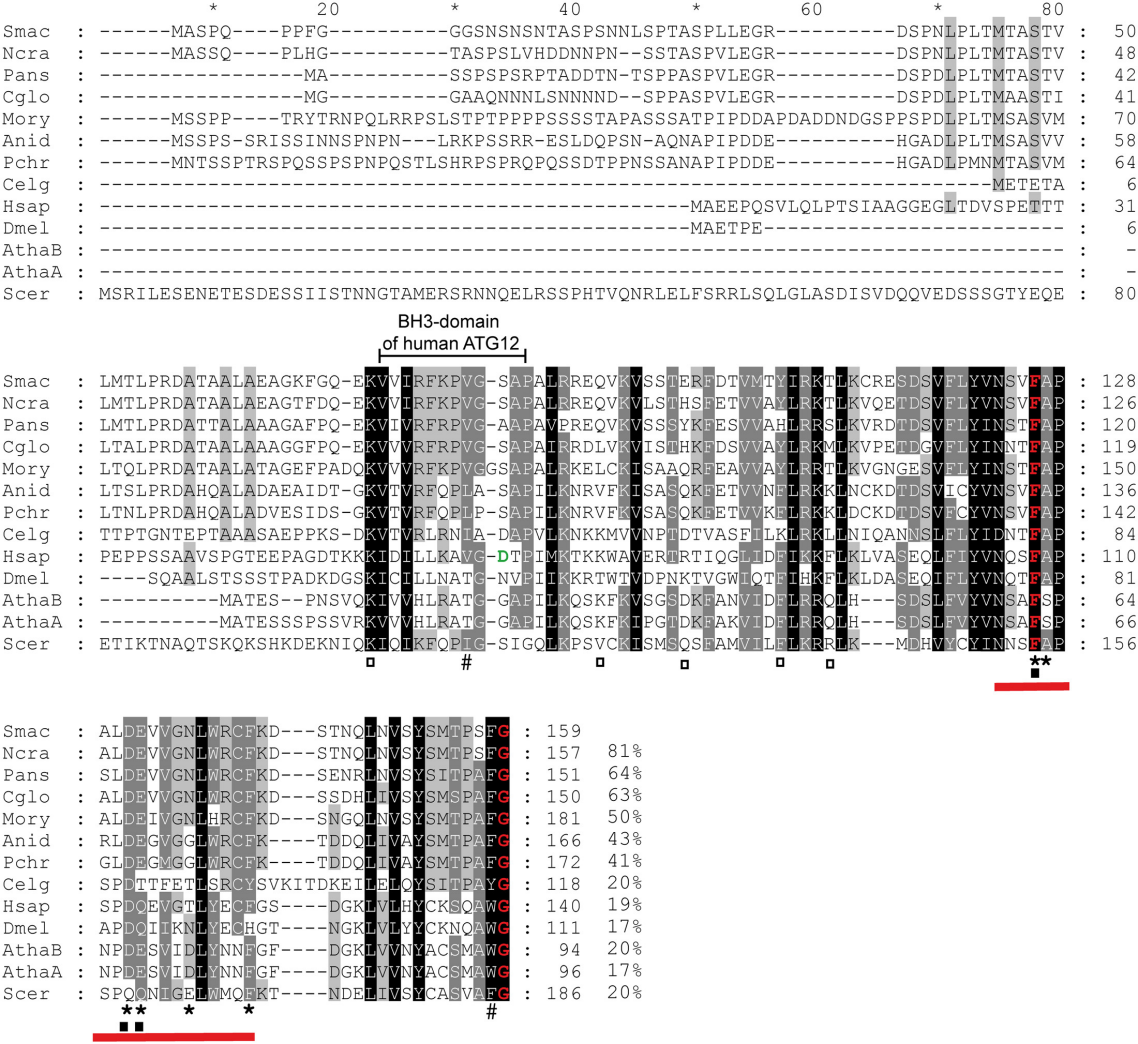
Multiple sequence alignment of ATG12 orthologs from fungi, plants and animals. ClustalX alignment was created using the following sequences: Smac [*S*. *macrospora*, Accession No. XP_003349162.1, excluding 56 C-terminal amino acids)], Ncra [*Neurospora crassa*, Q7S083.1], Pans [*Podospora anserina*, XP_001906089.1], Cglo [*Chaetomium globosum*, Q2GSG9.2], Mory [*Magnaporthe oryzae*, XP_368646.1], Anid [*Aspergillus nidulans*, Q5BCH0.2], Pchr [*Penicillium chrysogenum*, XP_002557636.1], Celg [*Caenorhabditis elegans*, CCD61524.1], Hsap [*Homo sapiens*, NP_004698.3], Dmel [*Drosophila melanogaster*, NP_648551.3], AthaB [*Arabidopsis thaliana*, Q9LVK3.1], AthaA [*A*. *thaliana*, Q8S924.1], Scer [*S*. *cerevisiae*, P38316]. Identical amino acids, which are conserved in all proteins, are shaded in black; residues conserved in at least 10 of 13 sequences are shaded in dark grey and residues conserved in at least eight sequences are shaded in light grey. The conserved C-terminal glycine residue for the covalent linkage to ATG5 and the conserved phenylalanine residue corresponding to Phe154 in the *S*. *cerevisiae* Atg12 is labelled in red [67], amino acids important for non-covalent interactions between ATG12 and ATG5 in *S*. *cerevisiae* according to Noda et al. [65] are marked by asterisks. Non-covalent contacts between ATG12 and ATG5 identified in the human homologs according to Otomo et al. [66] are marked by black squares. The red bar represents the turn—loop—alpha helix 2 segment (Asn105 –Phe123 of the human ATG12) which is associated with the interaction surface of ATG5 [66]. White squares mark residues of the human ATG12, which are important for binding of ATG3 [64], #, indicates residues of the non-canonical AIM of ATG12 involved in interaction with ATG8 [24]. The region of the BH3 domain identified in the human ATG12 homolog is indicated and the conserved aspartic acid residue is indicated in green. Amino-acid identity in % is given at the right margin.

A BLASTP search of predicted *S*. *macrospora* proteins [63] using *S*. *cerevisiae* ATG12 (P38316) as a query sequence identified a predicted protein of 215 amino acids encoded by the ORF *SMAC_06998* as the top hit (3e^-13^). Compared with ATG12 proteins from other organisms, the predicted protein SMAC_06998 has an extended C-terminal region but no glycine residue at the C-terminus (Fig A in S1 File). Automatic annotation predicted two introns in *SMAC_06998* (Fig A in S1 File); however RT-PCR amplification with primer pair 06998_f/06998_r and sequencing confirmed only splicing of the first intron, which leads to an earlier stop codon. The amplified cDNA encodes a protein of 159 amino acids with a conserved C-terminal glycine that shares a significant level of sequence similarity with ATG12 proteins of plants, animals that also have a conserved glycine at the C-terminus (Fig 1). The *S*. *macrospora* ATG12 homlog shares 81% and 50% identity to the previously described ATG12 proteins from *Neurospora crassa* and *Magnaporthe oryzae* [12,13], and only about 20% identity with homologs from *S*. *cerevisiae*, plants and animals (Fig 1). However, most of the residues involved in non-covalent interactions between ATG12 and ATG5, binding and conjugation of ATG3, or binding of ATG8 in the *S*. *cerevisiae* and the human ATG12 homologs are conserved in the *S*. *macrospora* ATG12 (Fig 1) [24,27,64–66]. Several residues within the BH3 domain of ATG12 homologs from mammals and non-mammalian vertebrates are also conserved. Similar to the *S*. *cerevisiae* and other fungal ATG12 homologs, a conserved aspartic acid residue (equivalent to D64 in human ATG12) that is required for binding of anti-apoptotic proteins is replaced by a serine residue at position 82 in the *S*. *macrospora* protein (Fig 1) [29].

In *S*. *cerevisiae*, direct protein-protein interactions between the UBL protein Atg12 and both E1/E2-like enzymes Atg7 and Atg3 have been reported [64,66,68]. To confirm that SmATG12 can interact with SmATG7 and SmATG3, yeast two-hybrid analysis was performed after cloning of *Smatg12*, *Smatg7* [37] and *Smatg3* (*S*. *macrospora* ORF *SMAC_05399*) into prey and bait vectors pGBKT7 and pGADT7.

**Fig 2:**
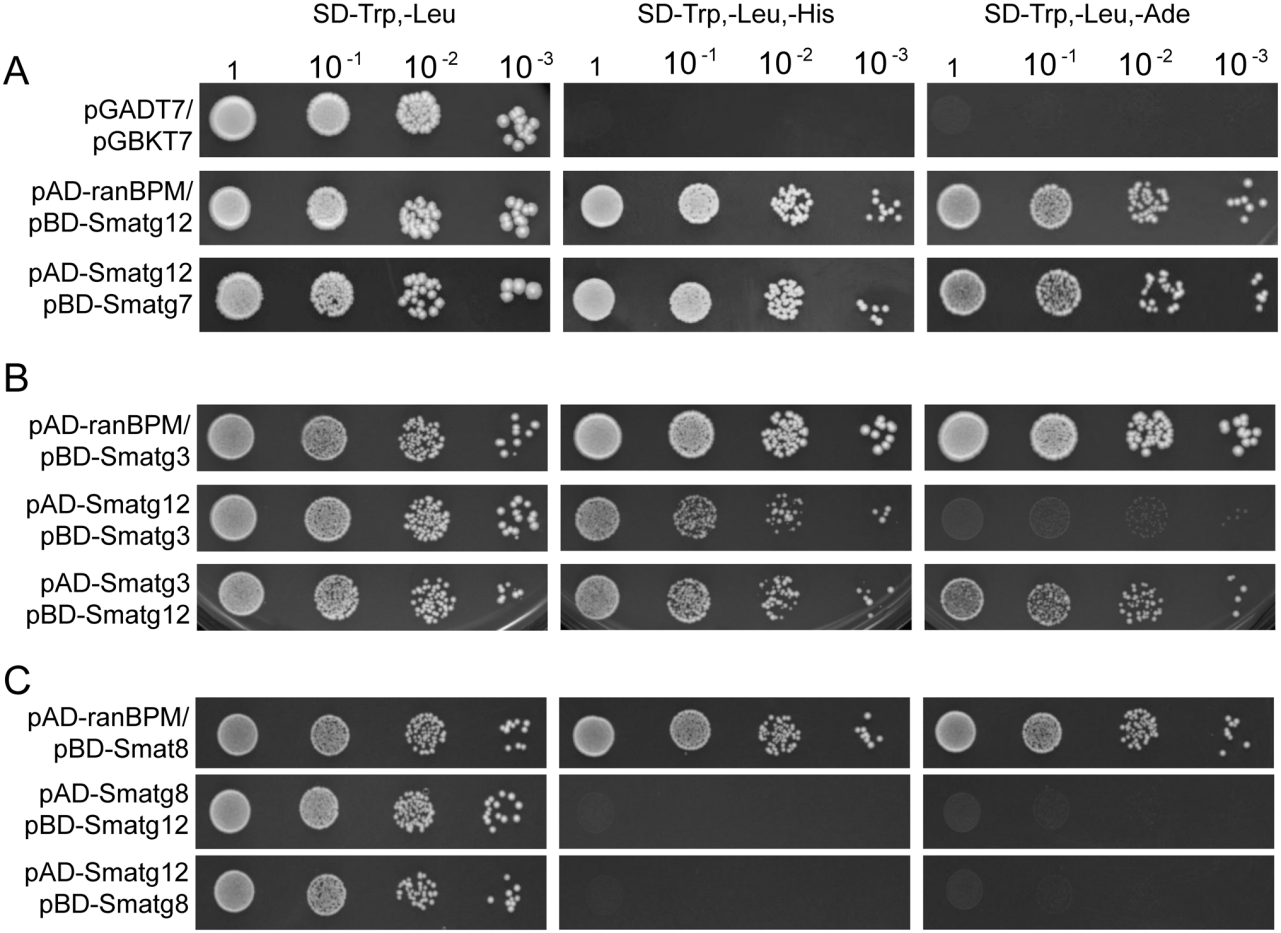
Yeast-two hybrid interaction of SmATG12 with SmATG7, SmATG8 and SmATG3. Full-length cDNAs of *Smatg12*, *Smatg7* and *Smatg3* were used to generate GAL4-DNA binding domain (BD) and activation domain (AD) plasmids. *Smatg8* two-hybrid vectors were previously described in Voigt and Pöggeler [34]. To select for the presence of both plasmids 20 μl of cells were spotted in serial delutions on SD medium lacking tryptophan and leucine (SD -Trp, -Leu) or to verify the interactions of the proteins on medium lacking additionally histidine or adenine (SD -Trp, -Leu, -His/-Ade). (A) SmATG12 and SmATG7 interacted only when SmATG7 was expressed as GAL4-BD fusion protein. (B) SmATG12 and SmATG3 interacted with each other as bait and prey proteins, respectively. (C) SmATG8 did not interact with SmATG12. Transformants carrying a bait plasmid and pAD-ranBPM [69] were used to confirm the appropriate expression of the bait proteins. Strains carrying empty plasmids pGADT7 and pGBKT7 served as negative control (A). Plates were incubated for 3–5 days at 30°C.

The resulting bait and prey plasmids were transformed into yeast strains Y187 and AH109, respectively, which were then mated, or both plasmids were co-transformed into strain AH109. Transactivation of pBD-derivatives was tested by mating with the AH109 strain carrying the empty vector pGADT7 (data not shown). As a positive control and to confirm expression of the proteins encoded by the bait plasmids, strains were mated with yeast strain AH109 containing pAD-ranBPM. RanBPM directly interacts with the GAL4-binding domain, which provides a method for confirming that the gene cloned into the bait vector is expressed appropriately [69]. The two-hybrid experiment clearly demonstrated an interaction between SmATG12 and both SmATG3 and SmATG7 (Fig 2A and 2B). SmATG12 and SmATG7 interacted only when SmATG7 was expressed as a GAL4-BD fusion protein (Fig 2A). Recently, Kaufmann et al. [24] demonstrated that the yeast ATG8-PE can interact directly with ATG12 via a non-canonical AIM in ATG12. However, interaction of SmATG12 and SmATG8 were not demonstrated in the yeast two-hybrid system (Fig 2C), although expression of SmATG8 and SmATG12 in yeast was verified by Western blot analysis (Fig B in S1 File).

Furthermore, we tested the functional conservation of SmATG12 and its yeast counterpart by a yeast complementation assay with the *Smatg12* cDNA expressed under the control of a *MET25* promoter in an *S*. *cerevisiae* atg12Δ null mutant. Rescue of autophagy in the *S*. *cerevisiae* mutant was monitored using an aminopeptidase I (API) maturation assay based on the autophagy-dependent maturation of the precursor proaminopetidase I (prAPI) to the mature enzyme (mAPI) [60]. In *S*. *cerevisiae*, API maturation relies on an intact UBL protein Atg12. However, the *Smatg12* gene was unable to complement the yeast atg12Δ mutant (Fig C in S1 File). In *S*. *cerevisiae*, API is delivered by the cytoplasm-to-vacuole-targeting pathway to the vacuole. So far it is not clear if this pathway exits in *S*. *macrospora*. In addition, we therefore monitored autophagy in the complemented mutant by the GFP-Atg8 proteolysis assay. When GFP-Atg8 is delivered to the lumen of the vacuole, the Atg8 part of the fusion protein is sensitive to degradation, whereas the GFP moiety is relative resistant to hydrolysis. In a Western blot with an anti-GFP antibody the appearance of free GFP can be used to monitor delivery of autophagosomal membranes to the vacuole [70]. The assay revealed that also no processing of GFP-Atg8 occurred in the atg12Δ yeast strain complemented with the *Smatg12* gene (Fig D in S1 File).

### Deletion of *Smatg12* impaired vegetative growth and fruiting-body development

**Fig 3:**
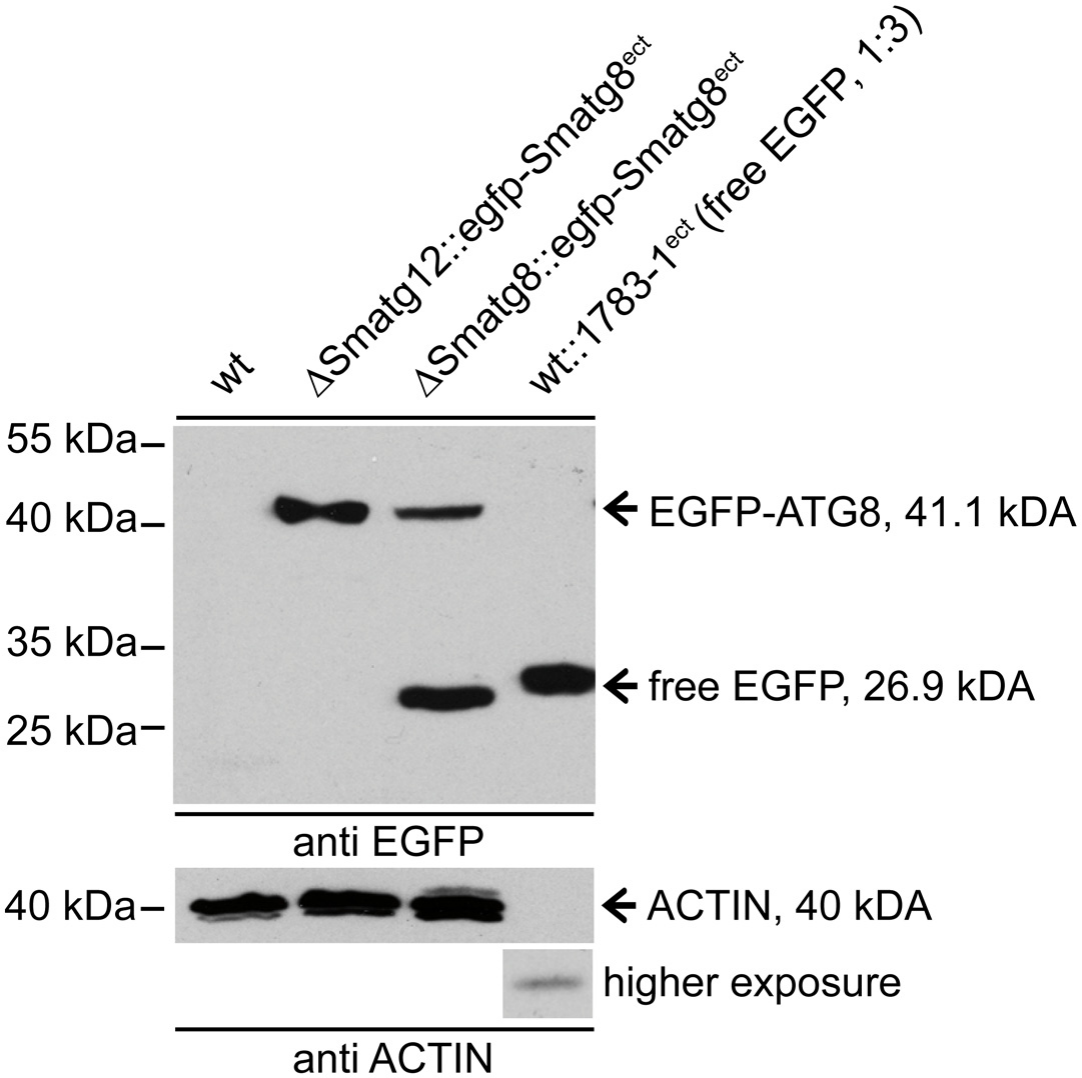
EGFP-SmATG8 protein degradation in the ΔSmatg12 strain compared to the corresponding complemented ΔSmatg8::egfp-Smatg8^ect^ strain. Protein crude extracts of *S*. *macrospora* wt, ΔSmatg12::egfp-Smatg8^ect^ and ΔSmatg8::egfp-Smatg8^ect^ strains expressing EGFP or EGFP-SmATG8 were separated on a 12% SDS-PAGE gel. The Western blot hybridization using an anti-EGFP antibody verified the degradation of the EGFP-SmATG8 fusion protein in the complemented ΔSmatg8 strain by accumulation of free EGFP, whereas in the ΔSmatg12 mutant the EGFP-SmATG8 fusion protein accumulated.

To analyze the effect of *Smatg12* on autophagy, vegetative growth and sexual development of *S*. *macrospora*, we generated a ΔSmatg12 knockout mutant for phenotypic characterization (Fig E in S1 File). The EGFP-ATG8 proteolysis assay in the *S*. *macrospora* ΔSmatg12 mutant revealed that vacuolar degradation of the fusion protein is inhibited, whereas in the complemented ΔSmatg8 strain proper vacuolar proteolysis of the fusion protein can be observed (Fig 3). Although *Smatg12* cannot complement the *S*. *cerevisiae* atg12Δ strain, this result suggests, that the isolated gene is an ortholog of the yeast ATG12.

**Fig 4:**
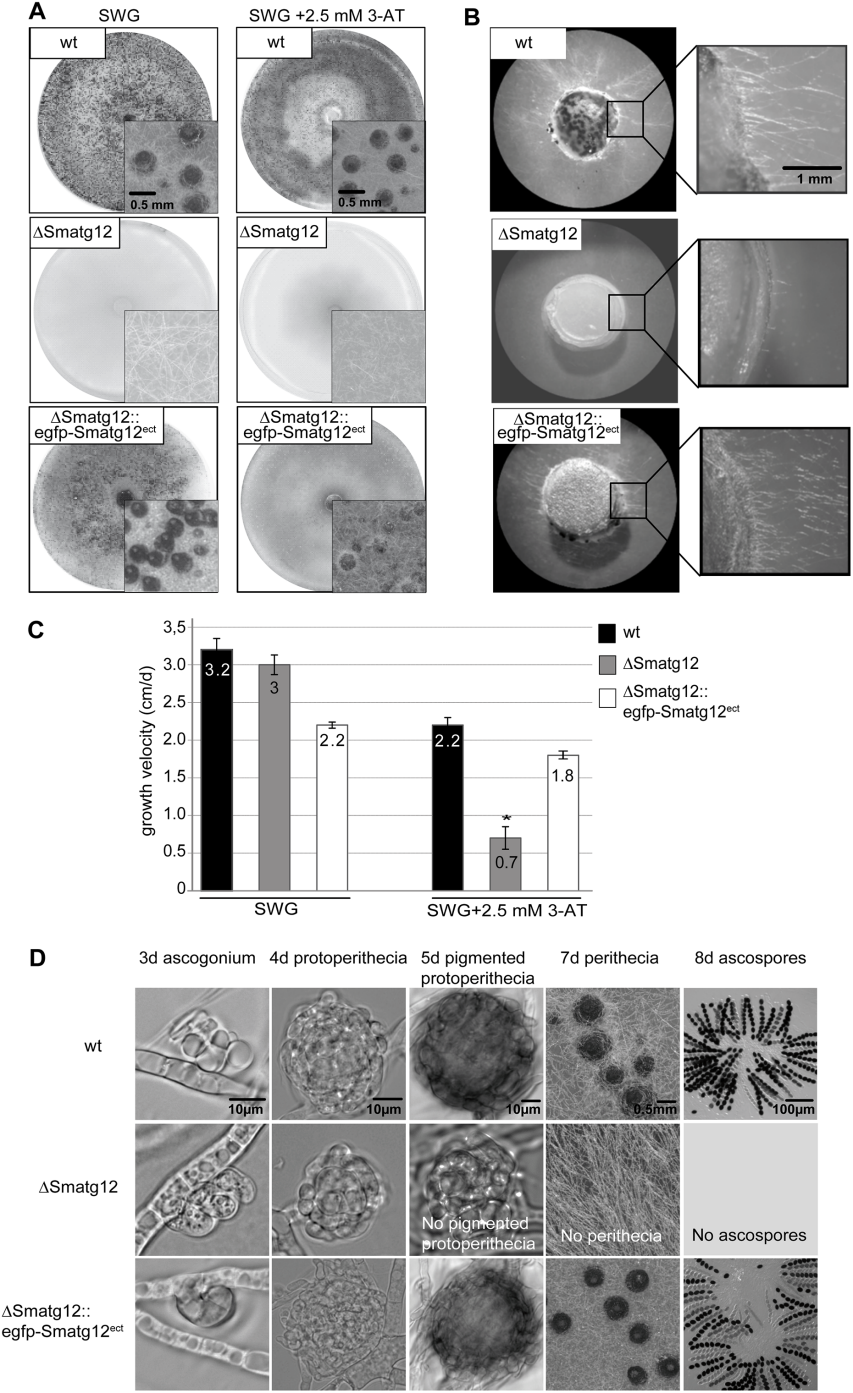
Phenotypic characterization of *S*. *macrospora* wt, *Smatg12* deletion and complementation strain. (A) Phenotype of wt, ΔSmatg12 and rescued strain ΔSmatg12::egfp-Smatg12^ect^ grown on SWG or SWG medium supplemented with 2.5 mM 3-AT in petri dishes. Insets show a detailed view of perithecia or sterile mycelium. The images were taken seven days post inoculation. (B) Foraging capacity of indicated strains. Agar plugs of 0.5-cm diameter were transferred into empty cell-culture plates (6 well, 17.2 ml) and incubated for 5 d at 27°C in a damp chamber before photographed. Scale bars as depicted. (C) The growth velocity of wt, ΔSmatg12 and the complemented strain was analyzed by measuring the growth velocity in cm/day in 30-cm race tubes. Growth rates on SWG medium shown are averages from 7 independent measurements of three independent experiments (n = 21), standard deviations are indicated by error bars. Asterisks indicate significant differences according to Student´s t-test (p<0.0000001). (D) Microscopic investigation of sexual development of ΔSmatg12 compared to wt and the complemented strain. Strains were grown on SWG medium. Expression of the EGFP-SmATG12 fusion construct complemented the sterile phenotype of ΔSmatg12. The wt and the complemented strain ΔSmatg12::egfp-Smatg12^ect^ form ascogonia at day 3, and protoperithecia at day 4 post inoculation. These develop to pigmented protoperithecia at day 5 and to mature perithecia at day 7. Sexual development of the mutant ΔSmatg12 is blocked at the stage of protoperithecia formation. ΔSmatg12 neither forms pigmented protoperithecia nor perithecia and ascospores. Scale bars as depicted.

In contrast to the wild-type (wt) strain and a complemented mutant strain (ΔSmatg12::egfp-Smatg12^ect^), the deletion mutant ΔSmatg12 did not form mature fruiting bodies when grown on solid SWG fructification medium or under histidine starvation conditions imposed using the drug 3-aminotriazole (3-AT) (Fig 4A). Prevention of autophagy is known to lead to the impairment of the foraging capability of filamentous ascomycetes [34,42], which describes the growth of filamentous fungi over a non-nutritious surface in order to reach nutrient-rich regions. The required nutrients are thought to be provided by autophagy taking place in the basal hyphae of mycelia [12,71]. To analyze the foraging capability of ΔSmatg12, wt and the complemented strain (ΔSmatg12::egfp-Smatg12^ect^), agar plugs were transferred into an empty cell-culture plate and incubated for 5 days. While the wt and the complemented strain underwent extensive mycelial growth, ΔSmatg12 was unable to grow over the inert plastic surface (Fig 4B). In addition, the deletion mutant displayed only a significant decrease in growth rate under histidine starvation conditions imposed by 3-AT (Fig 4C). In the complemented strain the growth defect was only partially complemented in comparison to the wt, probably due to an ectopic integration of the *egfp*-tagged *Smatg12*. Similarly, a slight growth defect of the complemented mutant could be observed under non-starvation conditions (SWG fructification medium), which might be also explained by ectopic integration of the *egfp*-tagged *Smatg12* wt copy.

Microscopic investigation of the fruiting-body development revealed that the mutant was arrested at the protoperithecia developmental stage and was unable to form mature fruiting bodies or ascospores (Fig 4A and 4D). Altogether, we analyzed five independent homokaryotic deletion mutants and two independent complemented mutants which displayed homogenous phenotypes. Thus, the observed phenotypes are associated with the deletion of the *Smatg12* gene.

### EGFP-SmATG12 localizes to phagophore assembly sites

**Fig 5:**
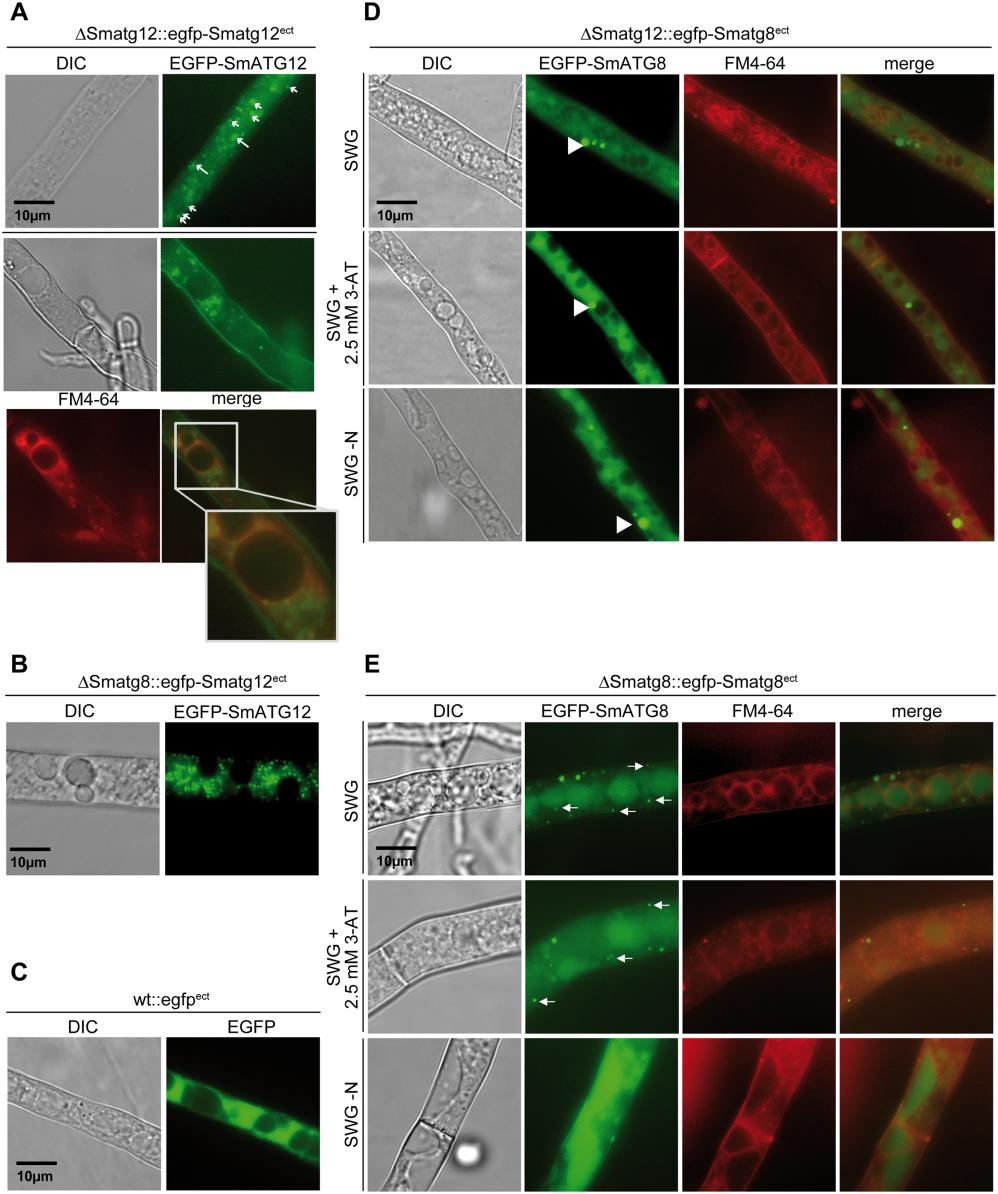
Fluorescence microscopic localization of EGFP-SmATG12 and EGFP-SmATG8. (A) Mutant ΔSmatg12 was transformed with plasmid pegfp-Smatg12. Expression of the EGFP-SmATG12 fusion construct complemented the sterile phenotype of ΔSmatg12. EGFP-SmATG12 localizes to the cytoplasm and at phagophore assembly sites indicated by small arrows and cup-shaped phagophores indicated by long arrows. Vacuolar membranes were stained using FM4-64. The merged picture shows a close up. (B) Localization of EGFP-SmATG12 in the ΔSmatg8 mutant [34]. The fluorescence signals are larger and more distinct than signals in the complemented mutant ΔSmatg8::egfp-Smatg8^ect^ (compare to E). (C) The fluorescence signal of free EGFP in the wt strain transformed with plasmid p1783-1 served as control. (D) When the mutant strain ΔSmatg12 expressed EGFP-SmATG8 (plasmid pRS-egfp-Smatg8 [34]), the fluorescence protein displays an equal diffused signal in the cytoplasm with large accumulating spots (arrow head) which are excluded from the vacuole. Vacuolar membranes were co-stained with FM4-64 and pictures were merged. (E) The previously constructed strain ΔSmatg8::egfp-Smatg8^ect^ [34] was used to compare the localization of EGFP-SmATG8 in the complemented ΔSmatg8 mutant. Autophagosomes are indicated by arrows. DIC, differential interference contrast; EGFP, enhanced green fluorescence protein. Autophagy was induced by the addition of 2.5 mM 3-AT to the SWG medium or by nitrogen starvation conditions (SWG-N, without KNO_3_ and arginine). Scale bars as depicted.

To investigate the *in vivo* localization of SmATG12, the deletion mutant ΔSmatg12 was transformed with plasmid pegfp-Smatg12. The ectopically integrated *egfp-Smatg12* fusion construct, under the control of the endogenous promoter, was able to complement the sterile phenotype of ΔSmatg12 (Fig 4). Fluorescence microscopy of the functionally expressed EGFP-SmATG12 fusion protein in the proliferating mycelium (24 h after inoculation) revealed localization in the cytoplasm as small discrete dots or cup-shaped structures that were presumed to be phagophore assembly sites or growing phagophores (Fig 5A). In the ΔSmatg8 mutant, the EGFP-SmATG12 fluorescence signals appeared larger and more distinct than that of the complemented ΔSmatg12 mutant (Fig 5B). To investigate the role of SmATG12 in autophagy, we examined the autophagy-mediated engulfment of EGFP-labeled SmATG8 into the vacuole. Transformation of pRS-egfp-Smatg8 [34] into ΔSmatg12 allowed us to follow the fluorescence signal of EGFP-labeled autophagosomes. In contrast to the complemented ΔSmatg8 mutant, in the ΔSmatg12 mutant EGFP-SmATG8 fluorescence was diffused throughout the cytoplasm, and large cytoplasmic aggregates were excluded from vacuoles, even in basal hyphae (Fig 5D). Vacuolar membranes were stained with the mebrane dys FM4-64. The localization did not change when we induced histidine starvation by 3-AT [34] or nitrogen limiting conditions. Fluorescence microscopy revealed the expected localization of EGFP-SmATG8 in vacuoles and in autophagosome-like structures (distinct spots in the cytoplasm) as well as in basal hyphal compartments in the lumen of the vacuoles in the complemented ΔSmatg8 (Fig 5E) [34]. These results indicated that macroautophagy was disrupted in the ΔSmatg12 mutant.

## Discussion

In contrast to ATG8 homologs, the function of the second UBL autophagy protein ATG12 has received much less attention in filamentous fungi [9]. We previously showed that the E1-like enzyme SmATG7, which presumably activates SmATG8 and SmATG12, is essential for viability in *S*. *macrospora*, whereas the UBL SmATG8 and its protease SmATG4 are involved in vegetative growth and sexual development [34,37]. In the present study, we investigated whether the second UBL SmATG12 also plays a role in sexual development and perithecia formation in this filamentous ascomycete.

### The conserved autophagy-associated protein SmATG12 was unable to rescue the *S*. *cerevisiae* atg12Δ mutant

Sequencing of the cDNA verified the presence of a C-terminal glycine in the deduced amino-acid sequence of *Smatg12* that is essential for the formation of an isopeptide bond with ATG5 [15] and was only reported to be absent in the *Plasmodium falciparum* ATG12 ortholog [72]. In addition, the carboxy-terminal region (aa 72–159) of SmATG12 revealed a high degree of sequence identity when compared with ATG12 homologs from *S*. *cerevisiae*, plants and animals (Fig 1). This region of SmATG12 was predicted to form an ATG12 ubiquitin-like domain (APG12_C, cd00196) by both the NCBI Conserved Domain Search tool (http://www.ncbi.nlm.nih.gov/Structure/cdd/wrpsb.cgi) [73] and InterProScan (http://www.ebi.ac.uk/interpro/) (PF04110) [74]. Within this domain, several amino acids and a turn-loop-alpha helix 2 segment, previously, shown in *S*. *cerevisiae* and *Homo sapiens* ATG12 to be important for non-covalent interactions with ATG5, were found to be conserved in SmATG12 [65,66]. In *S*. *macrospora*, *SMAC_08343* encodes a 366 aa ATG5 ortholog (Fig F in S1 File) that is larger than human or yeast ATG5 due to insertions, but it contains a conserved lysine residue (K218) that may form a conjugate with the C-terminal SmATG12 glycine residue (Fig F in S1 File). Yeast-two hybrid analysis revealed that SmATG12 can interact with the E1 enzyme SmATG7, which was previously shown to display characteristic features of E1-like enzymes (Fig 2A) [37]. In yeast and mammals, ATG7 activates ATG12 via C-terminal adenylation, and a conserved cysteine residue is subsequently covalently linked via a thioester bond to the C-terminal glycine residue [75]. A similar mechanism may therefore operate in *S*. *macrospora*.

After conjugation to ATG5, the ATG12~ATG5 conjugate acts in a complex with the coil-coiled protein ATG16 as an E3 ligase to conjugate ATG8 and PE. The ATG12~ATG5-ATG16 complex stimulates the transfer of ATG8 from the E2 enzyme ATG3 to PE [65,67,76]. Analysis of the human ATG12 protein structure showed that it binds a short peptide region of ATG3 [64]. Only some of these residues involved in this interaction are conserved in SmATG12 (Fig 1). However, yeast-two hybrid analysis confirmed the interaction of SmATG12 and SmATG3 (Fig 2B). In humans, a fraction of ATG3 was found to be conjugated to ATG12 via lysine 243 (K243) [27], which is also conserved in SmATG3 (Fig G in S1 File). Therefore, conjugation of SmATG3 and SmATG12 may also be of physiological relevance in *S*. *macrospora*.

Two residues which were shown to mimic a non-canonical AIM in ATG12 are conserved in SmATG12 (Fig 1) [24]. However, an interaction of SmATG12 and SmATG8 could not be demonstrated in the yeast-two hybrid analyses (Fig 2). This might be explained by non-efficient lipidation of SmATG8 in yeast.

Even though *S*. *macrospora* ATG12 has many conserved features, the *Smatg12* cDNA was unable to complement the *S*. *cerevisiae* atg12Δ mutant. Interestingly, this was also the case when *Arabidopsis thaliana* ATG12 orthologs were tested in *S*. *cerevisiae* [77]. We previously showed that *Smatg7*, *Smatg8* and *Smatg4* all complement the autophagy defects of their corresponding yeast mutants [34,37]. The amino-acid sequence identity shared between SmATG7, SmATG8 and SmATG4 and their *S*. *cerevisiae* homologs is 53%, 76% and 43%, respectively. In contrast, the sequence identity of SmATG12 and *S*. *cerevisiae* ATG12 is only 20%. In particular, the extended N-terminal region of SmATG12 is conserved only among filamentous ascomycetes (Fig 1). Therefore, structural differences might prevent functional complementation of the yeast mutant.

### *Smatg12* is required for sexual reproduction and normal vegetative growth

Similar to the autophagic core components SmATG8 and SmATG4, deficiency of SmATG12 led to a reduced vegetative growth rate and insufficient foraging under nutrient-limited conditions, as well as the arrest of fruiting-body development at the early protoperithecium formation stage under nutrient-rich conditions (Fig 4) [34]. In contrast to ΔSmatg8 and ΔSmatg4, a significant decrease in growth rate occurred only when starvation was induced by 3-AT (Fig 4C). Similarly, budding yeast *S*. *cerevisiae* and plant *atg12* mutants exhibited reduced viability only under starvation conditions [78–80].

Although deletion of *Smatg12* abolished fruiting-body formation and ascosporogenesis, even under nutrient-rich conditions, we were able to isolate a homokaryotic ΔSmatg12 knockout strain from the heterokaryotic primary transformant. This showed that in contrast to the E1 enzyme SmATG7, loss of SmATG12 was not detrimental to viability or essential for ascospore germination [37]. In *S*. *cerevisiae* and the fission yeast *Schizosaccharomyces pombe*, *atg12* deletion mutants also displayed disrupted ascosporogenesis, but only during nitrogen starvation [78,81]. Gene expression in *S*. *macrospora* undergoes significant differences during fruiting-body development [82], suggesting extensive *de novo* protein synthesis is required for fruiting-body maturation and ascospore formation. Defects in autophagy may result in a shortage of recycled amino acids and hence proteins that are critical for sexual development. Deletion of *atg12* genes in a filamentous fungus have only been reported for *N*. *crassa* and the plant pathogen *M*. *oryzae* [12,13]. The *N*. *crassa* Δatg12 mutant was able to initiate female development by producing ascogonia, and few small protoperithecia. Protoperithecia grafting experiments demonstrated that autophagy is required within the vegetative mycelium, because fertilized protoperithecia of *N*. *crassa* Δatg12 mutants grafted onto a wild-type mycelium were able to complete fruiting-body development and produced ascospores [12]. The *M*. *oryzae* ΔMoatg12 strain was nonpathogenic and defective in appressorium formation, but effects on fruiting-body development were not reported [13]. Atg12-defective plants develop normally but display premature senescence, produce less seeds under nutrient-rich conditions, and are hypersensitive to carbon and nitrogen starvation [79,80]. Similarly, knockdown of *atg12* in the protozoan parasite *Acanthamoeba castellanii* resulted in the inhibition of cyst formation [83]. Together, these results suggest that *Smatg12*-dependent autophagy may play an important role in *S*. *macrospora* development.

### Deletion of *Smatg12* abolishes delivery of EGFP labelled SmATG8 to the vacuole

The EGFP-SmATG8 proteolysis assay revealed that SmATG12 is required for proper autophagic degradation of an EGFP-SmATG8 fusion protein (Fig 3). Furthermore, fluorescence microscopy of the functional EGFP-SmATG12 fusion protein revealed localization in bright foci and cup-shaped structures that were presumed to be PAS and growing phagophores, respectively. EGFP-SmATG12 was dispersed throughout the cytoplasm (Fig 5A). In *S*. *cerevisiae* and mammalian cells, ATG5 and ATG16 are markers for phagophores (also known as isolation membranes) [25,26,84]. Both are components of the ATG12~ATG5-ATG16 complex that acts as an E3 enzyme for the efficient lipidation of ATG8 [20]. In a reconstituted *in vitro* system using giant unilamellar vesicles and recombinant proteins, in addition to its E3 function, the complex was shown to tether vesicular precursors during phagophore elongation [85].

In *S*. *cerevisiae*, the ATG12~ATG5-ATG16 complex is believed to be recruited to the PAS and to remain at the phagophore during expansion before detaching upon completion of autophagosome formation [26]. In accordance with these observations, we did not observe any labelling of mature autophagosomes or vacuoles in the strain expressing EGFP-SmATG12 (Fig 5A). Only a small portion of the ATG12~ATG5-ATG16 complex localizes to the PAS and the phagophore whereas the majority of the complex is reported to be localized to the cytoplasm [86]. Beside its function in autophagy and its association in the ATG12~ATG5-ATG16 complex, ATG12 carries out autophagy-independent roles. After conjugation to ATG3, it is involved in mitochondrial homeostasis, endosomal trafficking and apoptosis, whereas free ATG12 associates with anti-apoptotic Bcl-2 to promote mitochondrial apoptosis [27–29]. The exact localization of ATG12 during these processes remains to be determined.

When expressing EGFP-SmATG12 in an autophagy-deficient ΔSmatg8 mutant, the number of fluorescent foci appeared to increase and cup-shaped structures were not detected (Fig 5B). The enhanced localization of SmATG12 to these PAS-like structures may be explained by the blocking of phagophore extension due to the absence of SmATG8.

In the ΔSmatg12 deletion mutant, the EGFP-SmATG8 reporter was localized to a few small dot-like structures and large fluorescent aggregates in the cytoplasm (Fig 5D). This finding is in agreement with our previous findings in which expression of EGFP-SmATG8 in a ΔSmatg4 mutant resulted in the formation of large aggregates instead of small punctate autophagosomes [34]. Aggregates of a YFP-ATG8 reporter protein have been identified in a maize *atg12* mutant, and electron microscopy revealed these aggregates to be amorphous structures lacking a delineating membrane but containing the YFP-ATG8 reporter protein and ubiquitin [80]. In mice, ATG8 tended to aggregate in an autophagy-independent manner [87], and autophagosomes in other organisms failed to form when components of the ATG12~ATG5-ATG16 complex were deleted [15,22,84,88]. A fluorescence signal from EGFP-SmATG8 was not detected in the vacuoles of the ΔSmatg12 mutant, indicating that ATG8-mediated autophagy was affected (Fig 5D).

Our findings suggest that the fluorescent structures identified in the ΔSmatg12 mutant were EGFP-ATG8 aggregates that accumulated due to failed autophagosome formation. SmATG12-mediated autophagy appears to be essential for fruiting-body formation and the production of ascospores. Furthermore, SmATG12 was localized to phagophore structures. These results provide a better understanding of the ATG12 conjugation system in filamentous fungi.

## Supporting Information

S1 File Supporting Information. Bacterial and fungal strains used in this study (**Table A**). Plasmids used and generated in this study (**Table B**). Oligonucleotides used in this study **(Table C**). Protein sequence and nucleotide sequence of the verified CDS of *Smatg12 (SMAC_06998*). Intron sequence is indicated in red (**Fig A**). Verification of the protein expression of SmATG12 and SmATG8 in yeast two-hybrid transformants. The proteins SmATG12 (17 kDA) and SmATG8 (14 kDA) fused to the activation domain (AD) were detected with an anti-HA antibody and those fused to the DNA binding domain (BD) with an anti-myc antibody. Transformants carrying a single vector and the empty vector were used as positive and negative controls, respectively (**Fig B**). Complementation of the *Saccharomyces cerevisiae* atg12Δ autophagy mutant with the *S*. *macrospora Smatg12* gene. Complementation of the yeast strain atg12Δ with *Smatg12* under control of the yeast *MET25* promoter (pRS-met25-Smatg12) was analyzed using an aminopeptidase 1 (Ape1) maturation assay [60]. As positive controls, the *S*. *cerevisiae* wt strain BY4741 was transformed with the empty plasmid pRS426-met25 or atg12Δ was transformed with the yeast plasmid pRS-met25-Scatg12 expressing the endogenous *ATG12* gene. *S*. *cerevisiae* atg12Δ transformed with the empty vector pRS426-met25 served as negative control. Total cell extracts of an equal amount of yeast cells (0.2 OD_600_ equivalents of cells) of a growing culture under non-starvation conditions (-) or 4h nitrogen starved cells (+) were separated on 15% SDS-Page. After blotting, nitrocellulose membranes were probed with an anti-Ape1 antibody. Anti-actin antibody was used as loading control. prApe1, Ape1 precursor; mApe1, mature Ape1 (**Fig C**). Proteolytic cleavage of GFP-ScATG8. The wt, atg12Δ and the putative complementation strains atg12Δ+pRS-met25-Scatg12 and atg12Δ+pRS-met25-Smatg12 were transformed with pRS315-GFP-ATG8 (ScATG8). The GFP-ScATG8 protein was detected with an anti-GFP antibody and has an expected size of 40 kDA. The proteolytic cleavage of the GFP-ScATG8 fusion protein can be detected by the free GFP signal at 26 kDA (**Fig D**). Construction of a ΔSmatg12 mutant. (A) Schematic illustration of the *S*. *macrospora Smatg12* locus *SMAC_06998* (black arrow) with neighboring genes *SMAC_06997* and *SMAC_06999* and the derived ΔSmatg12 knockout locus. Gene replacement was reached by integration of an *hph*-deletion cassette at the respected gene locus (grey arrow) by homologous recombination. The intron is indicated as white box. Positions of primers used for knockout plasmid construction and verification of the successful homologous integration at the *Smatg12* locus are marked with small arrows. Sizes of the corresponding PCR fragments are indicated. *hph*, hygromycin resistance; P*trpC*, *Aspergillus nidulans trpC* promoter. (B) PCR analysis for verification of the integration of the *hph*-deletion cassette at the desired *Smatg12* gene locus in comparison to the wild type (wt). The positions of primers and expected fragment sizes indicated in (A) could be detected. (C) Southern hybridization for verification of the ΔSmatg12 strain. Genomic DNA from *S*. *macrospora* wt and the ΔSmatg12 deletion mutant was digested with *Bgl*I and hybridized with the 300-bp probe indicated in (A), which covered the promoter region of *Smatg12*. The expected fragments of 3398 bp for the wt and 4230 bp for the ΔSmatg12 deletion strain could be detected (**Fig E**). Multiple sequence alignment of ATG5 orthologs from *Homo sapiens*, *Saccharomyces cerevisiae* and *Sordaria macrospora*. ClustalX alignment was created using the following sequences: Scer [Accession No. *S*. *cerevisiae*, DAA11286.1], Hsap [*H*. *sapiens*, Q9H1Y0.2] and Smac [*S*. *macrospora*, XP_003347373.1]. Amino acids, which are conserved in all proteins, are shaded in black; residues conserved in two of three sequences are shaded in grey. The conserved lysine residue (Smac, Lys 218, Scer Lys 149, Hsap Lys 130) which forms the conjugate with the C-terminal SmATG12 glycine residue is marked in red (**Fig F**). Multiple sequence alignment of ATG3 orthologs from *Sordaria macrospora*, *Saccharomyces cerevisiae* and *Homo sapiens*. ClustalX alignment was created using the following sequences: Smac [*S*. *macrospora*, Accession No. F7VSU2], Scer [*S*. *cerevisiae*, EWG83474.1], and Hsap [*H*. *sapiens*, AAH02830.1]. Amino acids, which are conserved in all proteins, are shaded in black; residues conserved in two of three sequences are shaded in grey. The conserved lysine residue (Smac, Lys 216, Scer Lys 212, Hsap Lys 243) which forms the conjugate with the C-terminal ATG12 glycine residue is marked in red (**Fig G**). Detailed description of yeast complementation (**Method A**). Construction of yeast two hybrid vectors and verification of the expression of GAL4 fusion proteins (**Method B**).
(PDF)
