# Supplementary material for: Autophagy-Associated Protein SmATG12 Is Required for Fruiting-Body Formation in the Filamentous Ascomycete Sordaria macrospora
Source: PLoS One. 2016 Jun 16;11(6):e0157960. doi: 10.1371/journal.pone.0157960 (PMC4911038; doi:10.1371/journal.pone.0157960)
Supplement: S1 File — Bacterial and fungal strains used in this study (Table A). Plasmids used and generated in this study (Table B). Oligonucleotides used in this study (Table C). Protein sequence and nucleotide sequence of the verified CDS of Smatg12 (SMAC_06998). Intron sequence is indicated in red (Fig A). Verification of the protein expression of SmATG12 and SmATG8 in yeast two-hybrid transformants. The proteins SmATG12 (17 kDA) and SmATG8 (14 kDA) fused to the activation domain (AD) were detected with an anti-HA antibody and those fused to the DNA binding domain (BD) with an anti-myc antibody. Transformants carrying a single vector and the empty vector were used as positive and negative controls, respectively (Fig B). Complementation of the Saccharomyces cerevisiae atg12Δ autophagy mutant with the S. macrospora Smatg12 gene. Complementation of the yeast strain atg12Δ with Smatg12 under control of the yeast MET25 promoter (pRS-met25-Smatg12) was analyzed using an aminopeptidase 1 (Ape1) maturation assay [60]. As positive controls, the S. cerevisiae wt strain BY4741 was transformed with the empty plasmid pRS426-met25 or atg12Δ was transformed with the yeast plasmid pRS-met25-Scatg12 expressing the endogenous ATG12 gene. S. cerevisiae atg12Δ transformed with the empty vector pRS426-met25 served as negative control. Total cell extracts of an equal amount of yeast cells (0.2 OD600 equivalents of cells) of a growing culture under non-starvation conditions (-) or 4h nitrogen starved cells (+) were separated on 15% SDS-Page. After blotting, nitrocellulose membranes were probed with an anti-Ape1 antibody. Anti-actin antibody was used as loading control. prApe1, Ape1 precursor; mApe1, mature Ape1 (Fig C). Proteolytic cleavage of GFP-ScATG8. The wt, atg12Δ and the putative complementation strains atg12Δ+pRS-met25-Scatg12 and atg12Δ+pRS-met25-Smatg12 were transformed with pRS315-GFP-ATG8 (ScATG8). The GFP-ScATG8 protein was detected with an anti-GFP antibody and has an expected size of 40 kDA. [file pone.0157960.s001.pdf]

S1 Supporting Information

Autophagy-associated protein SmATG12 is required for fruiting-body formation in the filamentous ascomycete *Sordaria macrospora*

Antonia Werner[1], Britta Herzog[1], Stefan Frey[1], Stefanie Pöggeler[1,2,*]

Supplementary Material and Methods

S1 File. Supporting Information Bacterial and fungal strains used in this study (**Table A**). Plasmids used and generated in this study (**Table B**). Oligonucleotides used in this study **(Table C**). Protein sequence and nucleotide sequence of the verified CDS of *Smatg12 (SMAC_06998*). Intron sequence is indicated in red (**Figure A**). Verification of the protein expression of SmATG12 and SmATG8 in yeast two-hybrid transformants. The proteins SmATG12 (17 kDA) and SmATG8 (14 kDA) fused to the activation domain (AD) were detected with an anti-HA antibody and those fused to the DNA binding domain (BD) with an anti-myc antibody. Transformants carrying a single vector and the empty vector were used as positive and negative controls, respectively (**Figure B**). Complementation of the *Saccharomyces cerevisiae* atg12Δ autophagy mutant with the *S. macrospora Smatg12* gene. Complementation of the yeast strain atg12Δ with *Smatg12* under control of the yeast *MET25* promoter (pRS-met25-Smatg12) was analyzed using an aminopeptidase 1 (Ape1) maturation assay (Harding et al., 1995). As positive controls, the *S. cerevisiae* wt strain BY4741 was transformed with the empty plasmid pRS426-met25 or atg12Δ was transformed with the yeast plasmid pRS-met25-Scatg12 expressing the endogenous *ATG12* gene. *S. cerevisiae* atg12Δ transformed with the empty vector pRS426-met25 served as negative control. Total

cell extracts of an equal amount of yeast cells (0.2 OD_{600} equivalents of cells) of a growing culture under non-starvation conditions (-) or 4h nitrogen starved cells (+) were separated on 15 % SDS-Page. After blotting, nitrocellulose membranes were probed with an anti-Ape1 antibody. Anti-actin antibody was used as loading control. prApe1, Ape1 precursor; mApe1, mature Ape1 (**Figure C**). Proteolytic cleavage of GFP-ScATG8. The wt, atg12Δ and the putative complementation strains atg12Δ+pRS-met25-Scatg12 and atg12Δ+pRS-met25-Smatg12 were transformed with pRS315-GFP-ATG8 (ScATG8). The GFP-ScATG8 protein was detected with an anti-GFP antibody and has an expected size of 40 kDA. The proteolytic cleavage of the GFP-ScATG8 fusion protein can be detected by the free GFP signal at 26 kDA (**Figure D**). Construction of a ΔSmatg12 mutant. (A) Schematic illustration of the *S. macrospora Smatg12* locus *SMAC_06998* (black arrow) with neighboring genes *SMAC_06997* and *SMAC_06999* and the derived ΔSmatg12 knockout locus. Gene replacement was reached by integration of an *hph*-deletion cassette at the respected gene locus (grey arrow) by homologous recombination. The intron is indicated as white box. Positions of primers used for knockout plasmid construction and verification of the successful homologous integration at the *Smatg12* locus are marked with small arrows. Sizes of the corresponding PCR fragments are indicated. *hph*, hygromycin resistance; P*trpC*, *Aspergillus nidulans trpC* promoter. (B) PCR analysis for verification of the integration of the *hph*-deletion cassette at the desired *Smatg12* gene locus in comparison to the wild type (wt). The positions of primers and expected fragment sizes indicated in (A) could be detected. (C) Southern hybridization for verification of the ΔSmatg12 strain. Genomic DNA from *S. macrospora* wt and the ΔSmatg12 deletion mutant was digested with *Bgl*I and hybridized with the 300-bp probe indicated in (A), which covered the promoter region of *Smatg12*. The expected fragments of 3398 bp for the wt and 4230 bp for the ΔSmatg12 deletion strain could be detected (**Figure E**). Multiple sequence alignment of ATG5 orthologs from *Homo sapiens*,

Saccharomyces cerevisiae and *Sordaria macrospora*. ClustalX alignment was created using the following sequences: Scer [Accession No. *S. cerevisiae*, DAA11286.1], Hsap [*H. sapiens*, Q9H1Y0.2] and Smac [*S. macrospora*, XP_003347373.1]. Amino acids, which are conserved in all proteins, are shaded in black; residues conserved in two of three sequences are shaded in grey. The conserved lysine residue (Smac, Lys 218, Scer Lys 149, Hsap Lys 130) which forms the conjugate with the C-terminal SmATG12 glycine residue is marked in red (**Figure F**). Multiple sequence alignment of ATG3 orthologs from *Sordaria macrospora*, *Saccharomyces cerevisiae* and *Homo sapiens*. ClustalX alignment was created using the following sequences: Smac [*S. macrospora*, Accession No. F7VSU2], Scer [*S. cerevisiae*, EWG83474.1], and Hsap [*H. sapiens*, AAH02830.1]. Amino acids, which are conserved in all proteins, are shaded in black; residues conserved in two of three sequences are shaded in grey. The conserved lysine residue (Smac, Lys 216, Scer Lys 212, Hsap Lys 243) which forms the conjugate with the C-terminal ATG12 glycine residue is marked in red (**Figure G**). Detailed description of yeast complementation (**Method A**). Construction of yeast two hybrid vectors and verification of the expression of GAL4 fusion proteins (**Method B**).

Tables

Table A. Bacterial and fungal strains used in this study.

Strain	Genotype	Source
Escherichia coli		
MACH1	*ΔrecA1398, endA1, tonA, Φ80ΔlacM15, ΔlacX74, hsdR,* (rK- mK+)	Invitrogen (Carlsbad, USA)
Saccharomyces cerevisiae		
PJ69-4A	*MAT***a***, trp1-901, leu2–311,; ura3–5,; his3–200, ga14Δ, ga18OΔ LYS2::GALl-HIS3, GAL2-ADE2, met2::GAL7-lacZ*	(James et al., 1996)
AH109	*Mat***a***; trp1-901; leu2-3, 112;ura3-52; his3-200; ade2-101;gal4Δ, gal80Δ; lys2::Gal1UAS-Gal1TATAhis3; Gal2UASGal2TATA ade2; ura3::Mel1UAS-Mel1TATA lacZ; Mel1*	Clontech laboratories inc. (Saint-Germain-en-Laye, France)
Y187	*Matα, ura3-52, his3-200, ade2-101, trp1-901, leu2-3, 112, gal4Δ, metΔ, gal80Δ, Mel1, ura3::Gal1UAS-Gal1TATA lacZ*	Clontech laboratories inc. (Saint-Germain-en-Laye, France)
BY4741	*MAT***a***, his3Δ1, leu2Δ0, ura3Δ0, met15Δ0*	EuroScarf
Y03357 (atg12Δ)	*BY4741, Mat***a***, his3Δ1, leu2Δ0, met15Δ0, ura3Δ0, YBR217w::kanMX4*	EuroScarf
Sordaria macrospora		
S48977	wt	U. Kück, Bochum
S23442	mutation in *fus1-1* gene; brownish ascospores	(Nowrousian et al., 2012)
Δku70	Δku70*::nat*^{R}	(Pöggeler and Kück, 2006)

ΔSmatg12	ΔSmatg12*::hyg*^{R}; ssi; sterile	This study
ΔSmatg12::egfp-Smatg12^{ect}	Δ*Smatg12::hyg*^{R}; *egfp-Smatg12*^{ect}; *nat*^{R}; ssi	This study
ΔSmatg12::egfp-Smatg8^{ect}	Δ*Smatg12::hyg*^{R}; *egfp-Smatg8*^{ect}; *nat*^{R}; ssi	This study
ΔSmatg8::egfp-Smatg12^{ect}	Δ*Smatg8::hyg*^{R}; *egfp-Smatg12*^{ect}; *nat*^{R}; ssi	This study
ΔSmatg8::egfp-Smatg8^{ect}	Δ*Smatg8::hyg*^{R}; *egfp-Smatg8*^{ect}; *nat*^{R}; ssi	(Voigt and Pöggeler, 2013)
wt::egfp^{ect}	*hyg*^{R}; *egfp*^{ect}; ssi	This study

nat^{R}, nourseothricin resistant; *hyg*^{R}, hygromycin resistant; ssi, single-spore isolate; ect, ectopical integrated.

Table B. Plasmids used and generated in this study.

Plasmid	Characteristics	Source
pCB1003	*amp*^{R}, *hph*	(Carroll et al., 1994)
pRS426	*URA*, *amp*^{R}	(Christianson et al., 1992)
pRSnat	*URA3*, *amp*^{R}, *nat*	(Klix et al., 2010)
pRS315-GFP-ATG8	*LEU*2, *amp*^{R}, *egfp-Scatg8* under control of endogenous promoter	(Mizushima et al., 2001)
p1783-1	*egfp* under control of *gpd* promoter and *trpC* terminator of *Aspergillus nidulans*, *hph*-cassette	(Pöggeler et al., 2003)
pSmatg12-KO	1058 bp of the 5'-flanking region and 700 bp of 3'-flanking region of *Smatg12* interrupted by the *hph*-cassette in pRS426	This study
pegfp-Smatg12	1058 bp of the 5'-flanking region of *Smatg12*; *egfp* exluding stop codon; *Smatg12* ORF without start codon and 1615 bp of 3'-flanking region of *Smatg12* in pRSnat	This study
pRS-egfp-Smatg8	1023 bp of the 5'-flanking region of *Smatg8*; *egfp* exluding stop codon; *Smatg8* ORF without start codon and 1021 bp of 3'-flanking region of *Smatg8* in pRSnat	(Voigt and Pöggeler, 2013)
pRS426-met25	*met25* promoter; *URA3*; *amp*^{R}	(Mumberg et al., 1994)
pRS-met25-Smatg12	*Smatg12* under control of *met25* promoter; *URA3*; *amp*^{R};	This study
pRS-met25-Scatg12	*Scatg12* under control of *met25* promoter; *URA3*; *amp*^{R};	This study
pGADT7	*LEU2*, *adh1*^{P}, *GAL4-AD*, *amp*^{R}	Clontech, 630442
pGBKT7	*TRP1*, *adh1*^{P}, *GAL4-BD*, *kan*^{R}	Clontech, 630443
pAD-ranBPM	*LEU2*, *GAL4-AD*, *amp*^{R}, *ran*BPM	(Tucker et al., 2009)
pAD-Smatg12	*LEU2*, *GAL4-AD*, *amp*^{R}, *Smatg12*	This study
pBD-Smatg12	*TRP1*, *GAL4-BD*, *kan*^{R}, *Smatg12*	This study

pAD-Smatg7	*LEU2*, *GAL4-AD*, *amp*^{R}, *Smatg7*	This study
pBD-Smatg7	*TRP2*, *GAL4-BD*, *kan*^{R}, *Smatg7*	This study
pAD-Smatg3	*LEU2*, *GAL4-AD*, *amp*^{R}, *Smatg3*	This study
pBD-Smatg3	*TRP2*, *GAL4-BD*, *kan*^{R}, *Smatg3*	This study

gpd promoter, glycerine aldehyde 3-phosphate dehydrogenase promoter from *A. nidulans*; *trpC* terminator, anthranilate synthase terminator from *A. nidulans*; *amp*^{R}, ampicillin resistance; *kan*^{R}, kanamycin resistance; *hph*, hygromycin B phosphotransferase, *nat*, nourseothricin N-acetyl transferase; *adh1*^{P}, *S. cerevisiae adh1* promoter. P, promoter.

Table C. Oligonucleotides used in this study.

Oligo	Sequence 5‘ -3‘
06998_f	ATGGCATCCCCACAACCCCC
06998_r	TCAGGTCCAGAGCAAGTAGT
hph-f	GTTAACTGATATTGAAGGAGCATTTTTGG
hph-r	GTTAACTGGTTCCCGGTCGGCATCTACTC
Atg12-gfp-5f	*GTAACGCCAGGGTTTTCCCAGTCACGACG*ACATACAGCCCAATTGATGG
Atg12-gfp-5r	*GTGAACAGCTCCTCGCCCTTGCTCACCAT*TTTGGGAAATTTAATGAGTG
Atg12-gfp-3f2	*TCACTCTCGGCATGGACGAGCTGTACAAG*GCATCCCCACAACCCCCATT
Atg12-gfp-3r	*GCGGATAACAATTTCACACAGGAAACAGC*CGGTGCTTTCGCCATGGTCG
GFP-f	ATGGTGAGCAAGGGCGAGGAGC
GFP-r	CTTGTACAGCTCGTCCATGCCGAGAGTG
Atg12_5f	*GTAACGCCAGGGTTTTCCCAGTCACGACG*ACATACAGCCCAATTGATGG
Atg12_5r	*CAAAAAATGCTCCTTCAATATCAGTTAAC*TTTGGGAAATTTAATGAGTG
Atg12_3f	*GAGTAGATGCCGACCGGGAACCAGTTAAC*TGCCGGGAGTCGGACAGCGT
Atg12_3r	*GCGGATAACAATTTCACACAGGAAACAGC*GAGTGCTGTTGATGTCGATG
Atg12_3D2	CCGTGCTGCTCACTTTGACC
Atg12_5D1	GTCGATTGTCGACATGGCCT
Atg12_3D1	AGCCTGGCCATGGTTGCCCT
Atg12_Hf	<u>GAATTC</u>CATGGCATCCCCACAACCCCC
Atg12_Hr	<u>GTCGAC</u>GCTTACCCAAAAGACGGGGTCA
Atg12_Hf2	<u>CATATG</u>GCATCCCCACAACCCCCATT
Atg12_Hr2	<u>GAATTC</u>GCTTACCCAAAAGACGGGGTCA
Atg12_PRf	TGTGCAGGCTCGCTAATAAC
Atg12_PRr	AAAGTACGGGCGTAATCACG
Atg12_Cf	<u>ACTAGT</u>ATGGCATCCCCACAACCCCC
Atg12_Cr	<u>GTCGAC</u>TTACCCAAAAGACGGGGTCA

Scatg12-f	ATACTCTAGAACTAGTATGAGTAGGATCCTAGAGAG
Scatg12-r	GCTTGATATCGAATTCTTAACCAAACGCTACGGATG
Atg3_Hf	CATATGAACTTCCTTAGATCAACCGC
Atg3_Hr	GAATTCGCTTAGACACCCATGGTGAAGT
Atg7_Hf	ACTAGTGAATTCATGGACCTCAAGTTTGCGACATTC
Atg7_Hr	GTCGACAAGCTTTCAAATCAATTCTCCCTCGCCTTC
tC1	GGTTTAGTCGTCCAGGCGGTG
h3	TACTCGCCGATAGTGGAAACC

Overhangs are labelled in italics. Restriction sides are underlined.

Figure legends

Figure A. Protein sequence and nucleotide sequence of the verified CDS of *Smatg12 (SMAC_06998*). Intron sequence is indicated in red.

Figure B. Verification of the protein expression of SmATG12 and SmATG8 in yeast two-hybrid transformants. The proteins SmATG12 (17 kDA) and SmATG8 (14 kDA) fused to the activation domain (AD) were detected with an anti-HA antibody and those fused to the DNA binding domain (BD) with an anti-myc antibody. Transformants carrying a single vector and the empty vector were used as positive and negative controls, respectively.

Figure C. Complementation of the *Saccharomyces cerevisiae* atg12Δ autophagy mutant with the *S. macrospora Smatg12* gene. Complementation of the yeast strain atg12Δ with *Smatg12* under control of the yeast *MET25* promoter (pRS-met25-Smatg12) was analyzed using an aminopeptidase 1 (Ape1) maturation assay (Harding et al., 1995). As positive controls, the *S. cerevisiae* wt strain BY4741 was transformed with the empty plasmid pRS426-met25 or atg12Δ was transformed with the yeast plasmid pRS-met25-Scatg12 expressing the endogenous *ATG12* gene. *S. cerevisiae* atg12Δ transformed with the empty vector pRS426-met25 served as negative control. Total cell extracts of an equal amount of yeast cells (0.2 OD_{600} equivalents of cells) of a growing culture under non-starvation conditions (-) or 4h nitrogen starved cells (+) were separated on 15 % SDS-Page. After blotting, nitrocellulose membranes were probed with an anti-Ape1 antibody. Anti-actin antibody was used as loading control. prApe1, Ape1 precursor; mApe1, mature Ape1.

Figure D. Proteolytic cleavage of GFP-ScATG8. The wt, atg12Δ and the putative complementation strains atg12Δ+pRS-met25-Scatg12 and atg12Δ+pRS-met25-Smatg12 were

transformed with pRS315-GFP-ATG8 (ScATG8). The GFP-ScATG8 protein was detected with an anti-GFP antibody and has an expected size of 40 kDA. The proteolytic cleavage of the GFP-ScATG8 fusion protein can be detected by the free GFP signal at 26 kDA.

Figure E. Construction of a ΔSmatg12 mutant. (A) Schematic illustration of the *S. macrospora Smatg12* locus *SMAC_06998* (black arrow) with neighboring genes *SMAC_06997* and *SMAC_06999* and the derived ΔSmatg12 knockout locus. Gene replacement was reached by integration of an *hph*-deletion cassette at the respected gene locus (grey arrow) by homologous recombination. The intron is indicated as white box. Positions of primers used for knockout plasmid construction and verification of the successful homologous integration at the *Smatg12* locus are marked with small arrows. Sizes of the corresponding PCR fragments are indicated. *hph*, hygromycin resistance; P*trpC*, *Aspergillus nidulans trpC* promoter. (B) PCR analysis for verification of the integration of the *hph*-deletion cassette at the desired *Smatg12* gene locus in comparison to the wild type (wt). The positions of primers and expected fragment sizes indicated in (A) could be detected. (C) Southern hybridization for verification of the ΔSmatg12 strain. Genomic DNA from *S. macrospora* wt and the ΔSmatg12 deletion mutant was digested with *Bgl*I and hybridized with the 300-bp probe indicated in (A), which covered the promoter region of *Smatg12*. The expected fragments of 3398 bp for the wt and 4230 bp for the ΔSmatg12 deletion strain could be detected.

Figure F. Multiple sequence alignment of ATG5 orthologs from *Homo sapiens, Saccharomyces cerevisiae* and *Sordaria macrospora*. ClustalX alignment was created using the following sequences: Scer [Accession No. *S. cerevisiae*, DAA11286.1], Hsap [*H. sapiens*, Q9H1Y0.2] and Smac [*S. macrospora*, XP_003347373.1]. Amino acids, which are conserved

in all proteins, are shaded in black; residues conserved in two of three sequences are shaded in grey. The conserved lysine residue (Smac, Lys 218, Scer Lys 149, Hsap Lys 130) which forms the conjugate with the C-terminal SmATG12 glycine residue is marked in red.

Figure G. Multiple sequence alignment of ATG3 orthologs from *Sordaria macrospora, Saccharomyces cerevisiae* and *Homo sapiens*. ClustalX alignment was created using the following sequences: Smac [*S. macrospora*, Accession No. F7VSU2], Scer [*S. cerevisiae*, EWG83474.1], and Hsap [*H. sapiens*, AAH02830.1]. Amino acids, which are conserved in all proteins, are shaded in black; residues conserved in two of three sequences are shaded in grey. The conserved lysine residue (Smac, Lys 216, Scer Lys 212, Hsap Lys 243) which forms the conjugate with the C-terminal ATG12 glycine residue is marked in red.

Methods

Method A. Detailed description of yeast complementation

To analyze functional conservation of *atg12* in *S. macrospora* and *S. cerevisiae* the aminopeptidase 1 (Ape1) maturation assay was carried out as described by Harding et al. (1995). The 480-bp *Smatg12* cDNA amplified with primer pair Atg12_Cf/Atg12_Cr having *Spe*I and *Sal*I overhangs, respectively, was expessed under the control of the yeast *MET25* promoter in pRS-met25-Smatg12 (Mumberg et al., 1994). Plasmid pRS-met25-Scatg12 was generated by cloning a 561-bp *Scatg12* fragment amplified from *S. cerevisiae* BY4741 wt gDNA into the *Eco*RI/*Spe*I-hydrolyzed plasmid pRS426-met25. In the next step, yeast strain Y03357 (atg12Δ) was transformed with plasmids pRS-met25-Smatg12 and pRS-met25-Scatg12. As negative controls, the *S. cerevisiae* wt strain BY4741 as well as the yeast atg12Δ deletion strain, were transformed with the empty vector pRS426-met25 (Mumberg et al., 1994). To test rescue of the Ape1 processing in the atg12Δ mutant, cells were grown over night in SD minimal medium and adjusted to OD_{600} = 1. One set of cells was used for protein extraction, while another set was grown for four hours in SD minimal medium lacking nitrogen (SD-N) to induce amino-acid starvation and, in turn, autophagy in *S. cerevisiae* (Harding et al., 1995). For protein extraction yeast cells were centrifuged (4000 rpm, 10 min, 4°C) and washed with cold water and re-suspended in 1 ml A. dest. H_2O. Subsequently, cell suspension was mixed with 150 µl lysis buffer (1.85 M NaOH, 7.5 % (v/v) β-mercaptoethanol) and incubated for 10 min on ice. 50 % TCA was added and the suspension was incubated for additional 10 min on ice. After centrifugation (4000 rpm, 10 min, 4°C), the cell pellet was washed twice with acetone (-20°C) and re-suspended in 100 µl Laemmli buffer (0.35 M Tris/HCl, 10.28 % SDS, 36 % glycerin, 5 % β-mercaptoethanol, 0.012 % bromophenol blue) and incubated at 90°C for 10 min.

Samples were centrifuged at 4000 rpm for 10 min at 4°C before separating 10 – 15 µl of the protein solution by 12 % sodium dodecyl sulfate polyacrylamide gel electrophoresis (SDS-PAGE) (Laemmli, 1970), followed by blotting onto a nitrocellulose membrane (GE Healthcare, Amersham, 10600003, Germany). For blocking, the membrane was incubated in TBST buffer (1 % 1M TrisHCl pH 8.0, 0.86 % NaCl, 0.05 % Tween 20) with 5 % skim milk for 1 h at room temperature. Immunodetection of aminopeptidase 1 (Ape1) was performed with a polyclonal rabbit anti-Ape1 antibody (kindly provided by M. Thumm, Göttingen, 1:2500) and as secondary antibody HRP-linked anti-rabbit antibody (1:2500, Invitrogen G21234,) solved in TBST buffer with skim milk (5 %) was applied. The calibration of the Western blot was performed by immunodetection with a monoclonal mouse anti-actin antibody (1:2500, Novus NB100-74340,) and secondary HRP-linked goat anti-mouse antibody (1:2500, Dianova, 115-035-003). Primary antibodies were incubated over night at 4°C and secondary antibodies for 1 h at room temperature. To monitor processing of EGFP-ATG8 an anti-EGFP direct HRP-linked antibody (1:4000, Acris GmbH R1091HRP,) was used. Detection was performed using the Immobilon Western Kit (Millipore, WBKLS0500) and X-ray films (Fujifilms) in an Optimax X-ray filmprocessor (Protec Gmbh & Co.KG, 1170-1-0000).

Method B. Construction of yeast two-hybrid vectors and verification of the expression of GAL4 fusion proteins

The prey plasmid pAD-Smatg12 was constructed by cloning an amplified cDNA fragment of *Smatg12* using primer pair Atg12_Hf2/Atg12_Hr2 with added restriction sites *Nde*I and *Eco*RI, respectively, into *Nde*I/*Eco*RI-linearized vector pGADT7. To generate the bait vector pBD-Smatg12, the *Smatg12* amplicon amplified with primer pair Atg12_Hf/Atg12_Hr with *Eco*RI and *Sal*I overhangs was cloned into the *Eco*RI/*Sal*I-linearized vector pGBKT7.

Plasmids pAD-Smatg7 and pBD-Smatg7 were constructed by amplifying a *Smatg7* cDNA fragment using primer pair Atg7_Hf/Atg7_Hr, which was ligated in *Eco*RI/*Hind*III-hydrolyzed pGADT7/pGBKT7 vector, respectively. Plasmids pAD-Smatg3 and pBD-Smatg3 were obtained by amplifying *Smatg3* with primer pair Atg3_Hf/Atg3_Hr from *S. macrospora* wt cDNA and inserting it as *Nde*I/*Eco*RI-fragment into hydrolyzed vectors pGADT7 and pGBKT7, respectively. The ligation reactions were conducted with T4-DNA ligase (Thermo Scientific) and the setup was arranged as follows: 1 µl T4-DNA ligase, 2 µl 10x T4-DNA ligation buffer, 1 µl hydrolyzed vector DNA and 16 µl PCR fragment (dialyzed). Reactions were incubated at RT for 30 minutes to 2 h. Plasmids pAD-Smatg8 and pBD-Smatg8 have been previously described in Voigt and Pöggeler (2013).

To verify expression of AD- and BD-fusion proteins, yeast cell cultures were grown at 30°C to midlog phase (OD_{600} 0.8). Harvested cells were washed in 500 µl ice-cold buffer b (100 mM Tris-HCl pH 7.5, 200 mM NaCl, 20 % glycerol, 5 mM EDTA) and lysed with glass beads in 500 µl of buffer b+ (+1x complete Protease Inhibitor Mixture, 0.5 % mercaptoethanol), and protein extracts were obtained via centrifugation at 13,000 rpm for 15 min. Proteins were denatured in SDS loading dye at 95°C for 15 min and subjected to SDS-PAGE followed by blotting onto a nitrocellulose membrane. The membranes were incubated with different antibodies as follows: SmATG12 and SmATG8 proteins fused to the activation domain in the pGADT7 vector were detected using a monoclonal mouse anti-HA antibody (diluted 1:3000, Sigma-Aldrich A2095), proteins fused to the binding domain in the pGBKT7 vector were detected using a monoclonal mouse anti-myc antibody (1:5000, Cell Signaling Technology 2279) and an anti-mouse HRP-linked secondary antibody (1:10000, Dianova, 115-035-003).

References

Carroll, A. M., et al., 1994. Improved vectors for selecting resistance to hygromycin. Fungal Genet Newslett. 41, 22.

Christianson, T. W., et al., 1992. Multifunctional yeast high-copy-number shuttle vectors. Gene. 110, 119-122.

Harding, T. M., et al., 1995. Isolation and characterization of yeast mutants in the cytoplasm to vacuole protein targeting pathway. J Cell Biol. 131, 591-602.

James, P., et al., 1996. Genomic libraries and a host strain designed for highly efficient two-hybrid selection in yeast. Genetics. 144, 1425-1436.

Klix, V., et al., 2010. Functional characterization of *MAT1-1*-specific mating-type genes in the homothallic ascomycete *Sordaria macrospora* provides new insights into essential and non-essential sexual regulators. Eukaryot. Cell. 9, 894-905.

Laemmli, U. K., 1970. Cleavage of structural proteins during the assembly of the head of bacteriophage T4. Nature. 227, 680-685.

Mizushima, N., et al., 2001. Dissection of autophagosome formation using Apg5-deficient mouse embryonic stem cells. The Journal of Cell Biology. 152, 657-668.

Mumberg, D., et al., 1994. Regulatable promoters of *Saccharomyces cerevisiae*: comparison of transcriptional activity and their use for heterologous expression. Nucleic Acids Research. 22, 5767-5768.

Nowrousian, M., et al., 2012. Whole-genome sequencing of *Sordaria macrospora* mutants identifies developmental genes. G3 (Bethesda). 2, 261-270.

Pöggeler, S., Kück, U., 2006. Highly efficient generation of signal transduction knockout mutants using a fungal strain deficient in the mammalian *ku70* ortholog. Gene. 378, 1-10.

Pöggeler, S., et al., 2003. Versatile EGFP reporter plasmids for cellular localization of recombinant gene products in filamentous fungi. Current Genetics. 43, 54-61.

Tucker, C. L., et al., 2009. A genetic test for yeast two-hybrid bait competency using RanBPM. Genetics. 182, 1377-9.

Voigt, O., Pöggeler, S., 2013. Autophagy genes *Smatg8* and *Smatg4* are required for fruiting-body development, vegetative growth and ascospore germination in the filamentous ascomycete *Sordaria macrospora*. Autophagy. 9, 33-49.

***Smatg12* verified CDS sequence**

ATGGCATCCCCACAACCCCCATTCGGAGGAGGATCCAACTCCAACTCCAACACCGCTTCC
CCTTCCAACAACCTCTCACCAACAGCCTCCCCCCTCCTCGAAGGCCGCGATTCGCCCAAC
CTACCACTAACCATGACCGCCTCCACGGTTCTGATGACGCTTCCGCGGGACGCCACCGCC
GCCCTCGCCGAAGCCGGGAAGTTTGGCCAGGAGAAAGTGGTGATACGCTTCAAACCGGTC
GGGTCGGCGCCCGCCCTCCGCAGGGAGCAGGTCAAAGTGAGCAGCACGGAAAGGTTCGAT
ACGGTGATGACGTACATAAGAAAGACGCTCAAGTGCCGGGAGTCGGACAGCGTGTTTTTG
TATGTGAATAGCGTATTTGCGCCGGCGCTGGATGAGGTTGTGGGGAATTTGTGGAGGgtg
agtttacctgggttcttctttcttggtgatcttgtctgggtgactatgatgctagggata
tttgagagtgtgtgagtgctgtgctgacgatgtgtatcatttgcagTGCTTTAAAGATTC
GACGAACCAGTTGAATGTTTCTTATTCGATGACCCCGTCTTTTGGGTAA

SmATG12 translation of verified *Smatg12* cDNA

MASPQPPFGGGSNSNSNTASPSNNLSPTASPLLEGRDSPNLPLTMTASTVLMTLPRDATA
ALAEAGKFGQEKVVIRFKPVGSAPALRREQVKVSSTERFDTVMTYIRKTLKCRESDSVFL
YVNSVFAPALDEVVGNLWRCFKDSTNQLNVSYSMTPSFG

S1, Fig. A

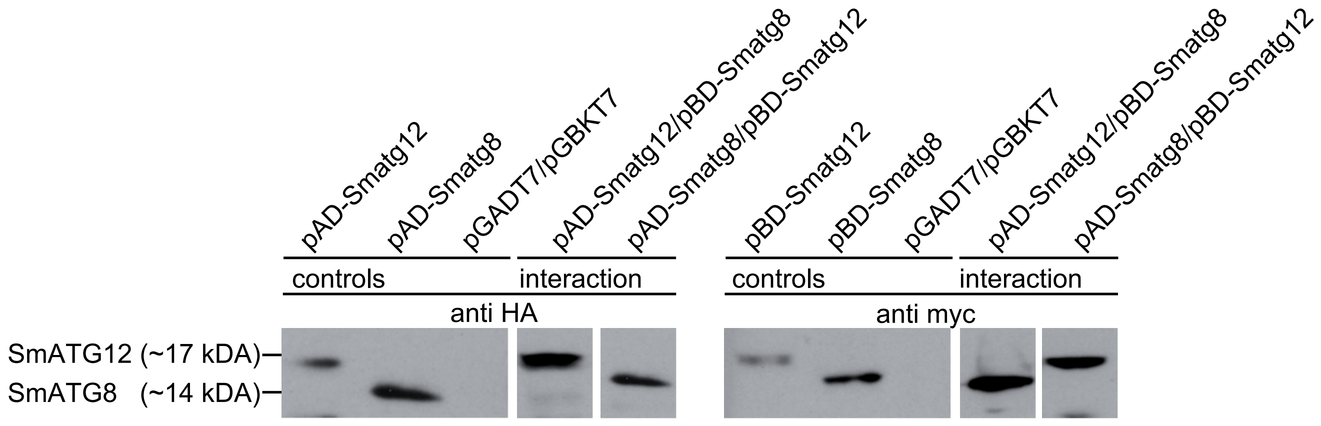
pAD-Smatg12
pAD-Smatg8
pGADT7/pGBKT7
pAD-Smatg12/pBD-Smatg8
pAD-Smatg8/pBD-Smatg12
pBD-Smatg12
pBD-Smatg8
pGADT7/pGBKT7
pAD-Smatg12/pBD-Smatg8
pAD-Smatg8/pBD-Smatg12
controls
interaction
anti HA
controls
interaction
anti myc
SmATG12 (~17 kDA)
SmATG8 (~14 kDA)

S1, Fig. B

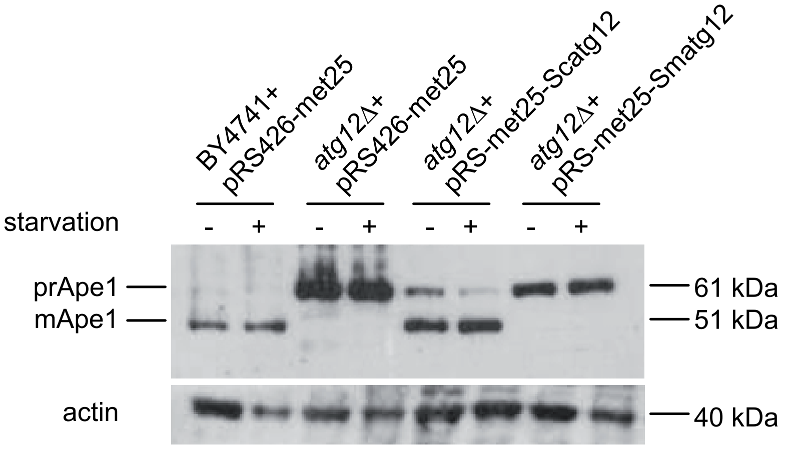
BY4741+
pRS426-met25
atg12Δ+
pRS426-met25
atg12Δ+
pRS-met25-Scatg12
atg12Δ+
pRS-met25-Smatg12
starvation
- + - + - + - +
prApe1
mApe1
actin
61 kDa
51 kDa
40 kDa

S1, Fig. C

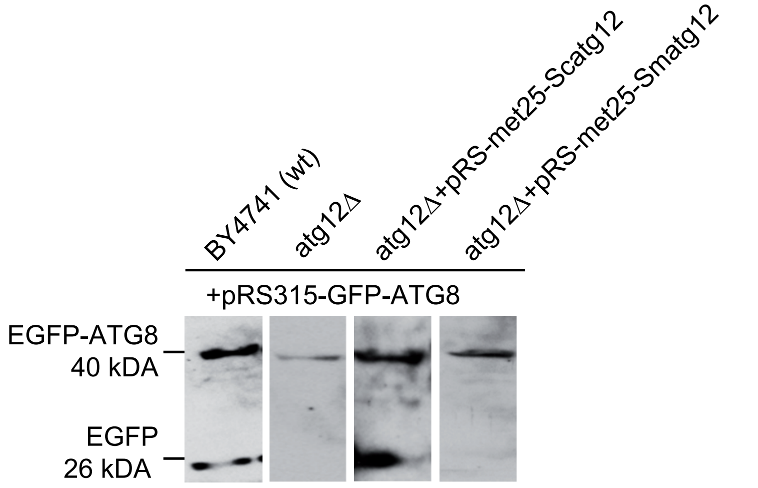
BY4741 (wt)
atg12Δ
atg12Δ+pRS-met25-Scatg12
atg12Δ+pRS-met25-Smatg12
+pRS315-GFP-ATG8
EGFP-ATG8
40 kDA
EGFP
26 kDA

S1, Fig. D

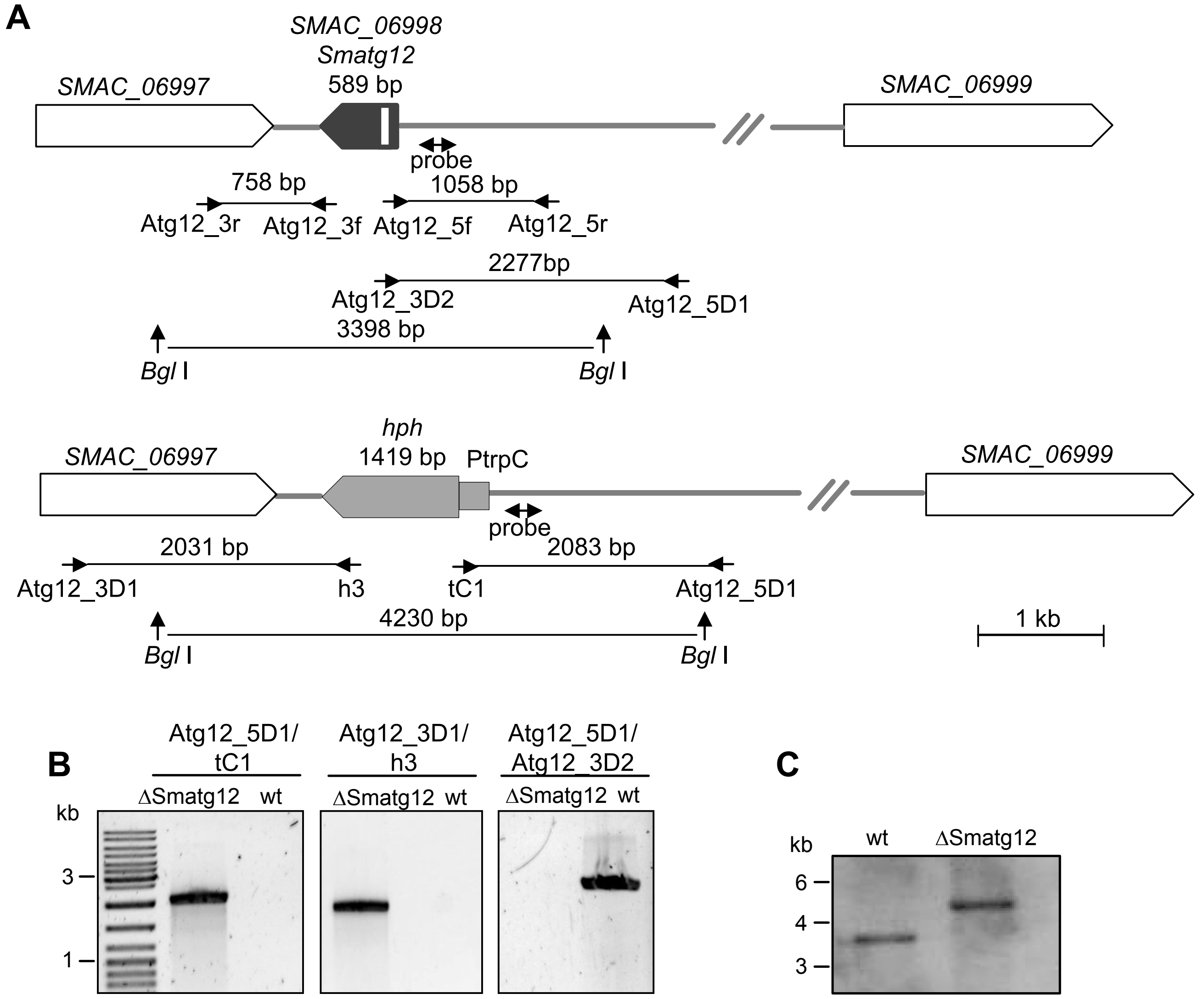
A
SMAC_06998
Smatg12
589 bp
SMAC_06997
SMAC_06999
probe
758 bp
1058 bp
Atg12_3r
Atg12_3f
Atg12_5f
Atg12_5r
2277bp
Atg12_3D2
Atg12_5D1
3398 bp
Bgl I
Bgl I
hph
1419 bp
PtrpC
SMAC_06997
SMAC_06999
probe
2031 bp
2083 bp
Atg12_3D1
h3
tC1
Atg12_5D1
4230 bp
Bgl I
Bgl I
1 kb
B
Atg12_5D1/
tC1
Atg12_3D1/
h3
Atg12_5D1/
Atg12_3D2
ΔSmatg12
wt
kb
3
1
C
kb
wt
ΔSmatg12
6
4
3

S1, Fig. E

```
                *        20         *        40         *        60         *        80
Scer : MNDIKQLLWNGELN---VLVSIDPSFLMKGSPREIAVLRIR--------------------------------------- :  38
Hsap : MTDDKDVLRDVWFG---RIPTCFTLYQDEITEREAEPYYLL--------------------------------------- :  38
Smac : MASPNPYSHSPQLPQREESPYQEDQYQSSLSFRSTYPFRSSRGAGSGTGLVLGHGHSPGFTSSLRGGGGGPASEHRYGSG :  80
       M d k  l    l      p     5q   3 Re  p r

                *       100         *       120         *       140         *       160
Scer : ----------VPRETYLVNYMPLIWNKIKSFLSFDPLTDSEKYFW----------FEHNKTPIPWNYP----VGVLFDCL :  94
Hsap : ----------LPRVSYLT----LVTDKVKKHFQKVMRQEDISEIW----------FEYEGTPLKWHYP----IGLLFDLL :  90
Smac : SGSRDGSHDDLPQRPLSSTTSSTVTPRHAPLLPKPKPTSIPETLWSLQIPLYITHQSHPKTPYICSVPRFSYLALLLPRL : 160
                 6Pr  yl     16t 4 k  l k   t      W          feh kTP  w yP    6q6Lfd L

                *       180         *       200         *       220         *       240
Scer : AGKSATFTTSFENQVKDVLTFLR---IHLVMGDSLPPTIIPIASSKTQAEKFWFHQWKQVCFILNGSSKAIMSLSVNEAR : 171
Hsap : ASSSALP----WNITVHFKSFPE---KDLLHCPSKDAIEAHFMSCMKEADALKHKSQVINEMQKKDHKQLWMGLQNDRFD : 163
Smac : TAYYGTPCSSFHHEEVHLRNLSVGLLVDLYQPSEMPWRLVVGDGDGWDICDTFMNSAKEADFIRNGNAKRIMGLSKEHTT : 240
       a  satp  sf n  vh   f      dL    s p       s    a      s k   fi ng  k iMgLs

                *       260         *       280         *       300         *       320
Scer : KFWGSVITRNFQDFIEI-------SNKISSSRPRHIPLIIQTSRTSGTFRISQPTISMTGVNPTLKDIEGDILD------ : 238
Hsap : QFW--AINRKLMEYPAE-------ENGFRYIPFRIYQTTTERPFIQKLFRPVAADGQLHTLGDLLKEVCPSAID------ : 228
Smac : ALWNAVQDNDYQAFTKVNTHLLNAPTALKNVPIRIYIPSSPPSSDNNKTQAAGSYRVMQTLVPPRGSNNRQALKSLLPAL : 320
        fW  vi r  q 5           n     p Riy      s     fr       6 t6 p lk     a6d

                *       340         *       360         *
Scer : VKEGINGNDVMVICQGIEIPWHMLLYDLYSKLRSFDGFLYITLVPIKGGDKASSEL : 294
Hsap : PEDGEKKN--QVMIHGIEPMLETPLQWLSEHLSYPDNFLHISIIPQPTD------- : 275
Smac : FPSSRDPVLANVILHGAPVPFLAPLEELMRDAAYPDGWLCLIVVLL---------- : 366
          g   n   V6 hGie p   pL  L   l ypDg5L 6 66p
```

S1 Fig. F

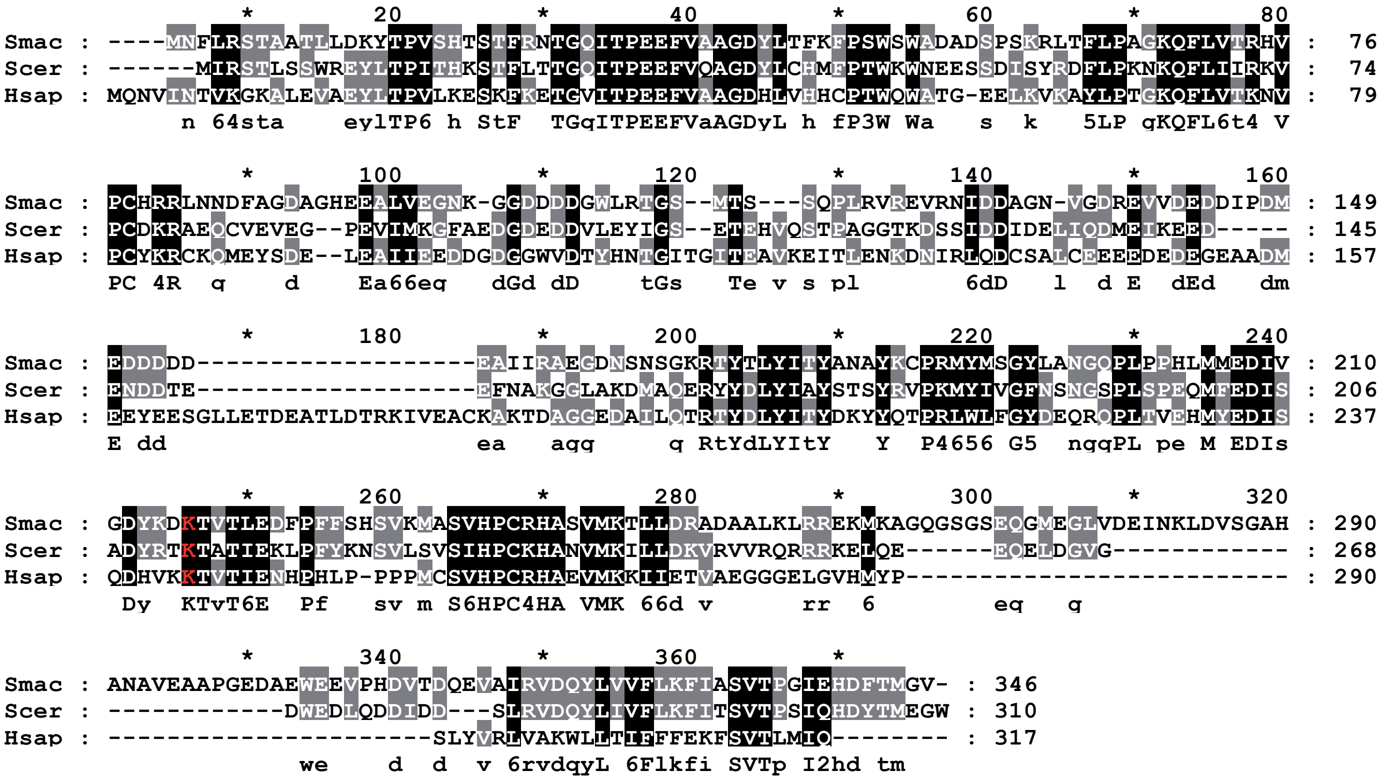

* 20 * 40 * 60 * 80
Smac : ----MNFLRSTAATLLDKYTPVSHTSTFRNTGQITPEEFVAAGDYLTFKFPSWSWADADSPSKRLTFLPAGKQFLVTRHV : 76
Scer : ------MIRSTLSSWREYLTPITHKSTFLTTGQITPEEFVQAGDYLCHMFPTWKWNEESSDISYRDFLPKNKQFLIIRKV : 74
Hsap : MQNVINTVKGKALEVAEYLTPVLKESKFKETGVITPEEFVAAGDHLVHHCPTWQWATG-EELKVKAYLPTGKQFLVTKNV : 79
n 64sta eylTP6 h StF TGqITPEEFVaAGDyL h fP3W Wa s k 5LP gKQFL6t4 V
* 100 * 120 * 140 * 160
Smac : PCHRRLNNDFAGDAGHEEALVEGNK-GGDDDDGWLRTGS--MTS---SQPLRVREVRNIDDAGN-VGDREVVDEDDIPDM : 149
Scer : PCDKRAEQCVEVEG--PEVIMKGFAEDGDEDDVLEYIGS--ETEHVQSTPAGGTKDSSIDDIDELIQDMEIKEED----- : 145
Hsap : PCYKRCKQMEYSDE--LEAIIEEDDGDGGWVDTYHNTGITGITEAVKEITLENKDNIRLQDCSALCEEEEDEDEGEAADM : 157
PC 4R q d Ea66eq dGd dD tGs Te v s pl 6dD l d E dEd dm
* 180 * 200 * 220 * 240
Smac : EDDDDD-------------------EAIIRAEGDNSNSGKRTYTLYITYANAYKCPRMYMSGYLANGQPLPPHLMMEDIV : 210
Scer : ENDDTE-------------------EFNAKGGLAKDMAQERYYDLYIAYSTSYRVPKMYIVGFNSNGSPLSPEQMFEDIS : 206
Hsap : EEYEESGLLETDEATLDTRKIVEACKAKTDAGGEDAILQTRTYDLYITYDKYYQTPRLWLFGYDEQRQPLTVEHMYEDIS : 237
E dd ea agg q RtYdLYItY Y P4656 G5 nqqPL pe M EDIs
* 260 * 280 * 300 * 320
Smac : GDYKDKTVTLEDFPFFSHSVKMASVHPCRHASVMKTLLDRADAALKLRREKMKAGQGSGSEQGMEGLVDEINKLDVSGAH : 290
Scer : ADYRTKTATIEKLPFYKNSVLSVSIHPCKHANVMKILLDKVRVVRQRRRKELQE------EQELDGVG------------ : 268
Hsap : QDHVKKTVTIENHPHLP-PPPMCSVHPCRHAEVMKKIIETVAEGGGELGVHMYP-------------------------- : 290
Dy KTvT6E Pf sv m S6HPC4HA VMK 66d v rr 6 eq g
* 340 * 360 *
Smac : ANAVEAAPGEDAEWEEVPHDVTDQEVAIRVDQYLVVFLKFIASVTPGIEHDFTMGV- : 346
Scer : ------------DWEDLQDDIDD---SLRVDQYLIVFLKFITSVTPSIQHDYTMEGW : 310
Hsap : ----------------------SLYVRLVAKWLLTIEFFEKFSVTLMIQ-------- : 317
we d d v 6rvdqyL 6Flkfi SVTp I2hd tm

S1 Fig. G
